# Modulating Local Electronic Structure via Cluster Engineering on Cobalt Phosphide for Efficient Water/Seawater Splitting

**DOI:** 10.1002/advs.202520390

**Published:** 2026-03-24

**Authors:** Cheng Gong, Fengying Pan, Pengpeng Zhang, Yuhan Xie, Yaojie Lei, Xiaobo Zheng, Dinushi Munasinghe Mudiyanselage, Hong Gao, Jinqiang Zhang, Yufei Zhao, Guoxiu Wang, Hao Liu

**Affiliations:** ^1^ Joint International Laboratory on Environmental and Energy Frontier Materials School of Environmental and Chemical Engineering Shanghai University Shanghai 200444 China; ^2^ School of Mathematical and Physical Sciences Faculty of Science University of Technology Sydney Broadway Ultimo Australia; ^3^ School of Chemistry The University of New South Wales Sydney NSW 2052 Australia

**Keywords:** alkaline natural seawater electrolysis, cluster engineering strategy, Fe‐O‐Co bridge, transition metal phosphides (TMPs)

## Abstract

Developing high‐performance, cost‐effective electrocatalysts for large‐scale water/seawater electrolysis is highly desirable, yet remains a significant challenge. Herein, oxidized iron nanocluster‐decorated cobalt phosphide (FeO_x_‐ACs/Co_x_P) is designed and explored for water splitting. These oxidized iron nanoclusters provide an optimal thermodynamic environment that enhances electron‐transfer capability due to the Fe‐O‐Co bridge at the interface. They donate electrons to nearby Co and P sites, tuning their coordination environment and enhancing electron‐transfer capability. As a result, FeO_x_‐ACs/Co_x_P exhibits outstanding oxygen evolution reaction (OER) performance with a low overpotential of 278 mV at 100 mA cm^−2^ and remarkable durability over 100 h at 100 mA cm^−2^. Mechanism investigation reveals the formation of high‐valence Co active center and the optimized adsorbate evolution mechanism (AEM) pathway for OER. The formation of *O is identified as the rate‐determining step (RDS) for FeO_x_‐ACs/Co_x_P with the lowest energy barrier. Moreover, FeO_x_‐ACs/Co_x_P shows promise for alkaline natural seawater electrolysis, requiring only 298 mV at 100 mA cm^−2^ with over 100 h stability. An anion‐exchange membrane water electrolysis (AEM‐WE) device using FeO_x_‐ACs/Co_x_P and Pt/C achieves a low voltage of 1.85 V at 500 mA cm^−2^. This work demonstrates the potential of a precise nanocluster engineering strategy in enhancing the electrocatalytic performance for water splitting.

## Graphical abstract

The heterostructure benefits from the high dispersion of FeO_x_ nanoclusters on the porous Co_x_P substrate, while the interfacial bonds Fe‐O‐Co enhance charge transport and redistribution, resulting in excellent oxygen evolution reaction performance and strong potential for practical applications.

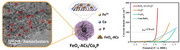

## 1 Introduction

Hydrogen energy has emerged as a viable replacement for non‐renewable energy due to its superior energy density, eco‐compatibility, and sustainable production potential.^[^ ^1^, ^2^ ^]^ Electrocatalytic water splitting stands out as a technologically feasible pathway for renewable hydrogen generation.^[^ ^3^, ^4^ ^]^ Despite decades of advancement, the large‐scale industrial deployment of water‐splitting technology remains constrained by high capital costs and challenges related to the electrolysis medium.^[^ ^5^, ^6^, ^7^ ^]^ The practical implementation of hydrogen production is further impeded by issues such as material corrosion, catalyst instability, and membrane degradation. Additionally, freshwater scarcity has prompted growing interest in seawater electrolysis as an alternative pathway for green hydrogen production.^[^ ^8^, ^9^ ^]^ However, the complex and corrosive nature of seawater poses significant obstacles, including accelerated electrode degradation and electrolyzer failure, raising critical concerns about the technological feasibility and economic viability of direct seawater splitting.^[^ ^10^ ^–^ ^15^ ^]^

Transition metal phosphides (TMPs) have emerged as compelling electrocatalyst candidates to the noble metal oxides (*e.g.*, IrO_2_, RuO_2_) due to their high natural abundance, superior charge transfer capability, and tunable d‐band configurations that demonstrate excellent OER catalytic efficiency.^[^ ^16^, ^17^, ^18^, ^19^, ^20^ ^]^ Benefiting from metal‐phosphorus (M‐P) covalent bonding that enhances metallic conductivity, TMPs such as FeP, Ni_2_P, and CoP demonstrate exceptional pre‐catalytic behavior in oxygen evolution reaction (OER) systems.^[^ ^21^, ^22^, ^23^, ^24^ ^]^ However, their practical implementation is hindered by nanoparticles (NPs) agglomeration tendencies and insufficient exposure of active sites, resulting in unsatisfied OER activity.^[^ ^25^, ^26^, ^27^ ^]^ In recent years, substantial research efforts have been devoted to developing engineering strategies to enhance the catalytic capability and stability of TMPs for OER. Morphological engineering strategies increase the surface area and mass transfer, and heteroatom doping (metal or non‐metal doping) can effectively modulate the electronic structure of TMPs, optimize the adsorption energy of active sites, and enhance their intrinsic catalytic activity. For instance, the introduction of Mo into CoP induced lattice strain and upshifts the d‐band center, thereby optimizing *OH adsorption during alkaline OER.^[^ ^28^ ^]^ Fe incorporation into Ni_5_P_4_ generated electron‐deficient Ni sites that enhanced OER activity by facilitating *OOH intermediate formation.^[^ ^29^ ^]^ Zn doping in CoP stabilized the phosphide lattice against oxidation while preserving metallic conductivity.^[^ ^30^ ^]^ These strategies have improved catalytic activity and stability; however, the influence of specific structural features on performance remains unclear, and their effectiveness under practical conditions is still limited.

Designing catalyst surfaces with components exhibiting atypical electronic configurations can induce cooperative charge redistribution, thereby enabling optimized interactions between active centers and reaction intermediates.^[^ ^31^, ^32^, ^33^, ^34^ ^]^ Interfacial engineering, particularly through nanocluster design, has emerged as a promising approach to leverage synergistic coupling between heterogeneous components. This approach induces interfacial electronic interactions and heterogeneous junctions, leading to modulation of the d‐band center of the clusters or metal substrates, which highly optimizes the adsorption energies of OER intermediates. The resulting structure also enables a continuous electron supply, helping to suppress excessive oxidation and corrosion of metal species in the electrolyte. Currently, most studies on nanoclusters have focused on noble‐metal‐based species, such as Ru, Ir, Pt, Pd, and Au, or materials derived from these noble metals. For example, Ru nanoclusters anchored on CoP/Ni_2_P heterostructure created strong metal‐support interactions. The Ru clusters donated electrons to CoP/Ni_2_P, downshifting its d‐band center and weakening *OOH binding.^[^ ^35^ ^]^ However, transition metal nanoclusters (*e.g.*, Fe, Co, Ni) represent a promising yet relatively underreported class of materials. This is largely due to the limitations of conventional solvothermal and related synthetic methods, which struggle to achieve surface‐enriched, transition metal‐based atomic interfaces. These nanoclusters offer unique opportunities for advancing fundamental understanding and catalytic performance.

Herein, through precise control of the interface engineering of surface clusters by the unique chemical vapor deposition (CVD) method, we demonstrate the rational designed iron oxide nanoclusters modified cobalt phosphide (FeO_x_‐ACs/Co_x_P) exhibit excellent performance for efficient water/seawater electrolysis. The constructed Fe‐O‐Co bridges at heterointerfaces create an optimized electronic environment that facilitates charge transfer and modulates the coordination geometry of cobalt centers, significantly enhancing OER kinetics. Comprehensive mechanistic investigations reveal the formation of high‐valent cobalt active sites and an optimized adsorbate evolution mechanism (AEM) pathway for the OER. The formation of *O species is identified as the rate‐determining step (RDS), with the FeO_x_‐ACs/Co_x_P demonstrating the lowest energy barrier among all control samples. As a result, FeO_x_‐ACs/Co_x_P exhibits a low overpotential of 278 mV at 100 mA cm^−2^ and outstanding stability for over 100 h at the same current density. In addition to pure water, it also delivers high current densities with excellent durability in seawater environments. This work provides fundamental insights into nanocluster‐mediated electronic regulation for developing cost‐effective electrocatalysts for large‐scale hydrogen production.

## 2 Results and Discussion

### 2.1. Compositional and Structure Characterization

**Figure 1:**
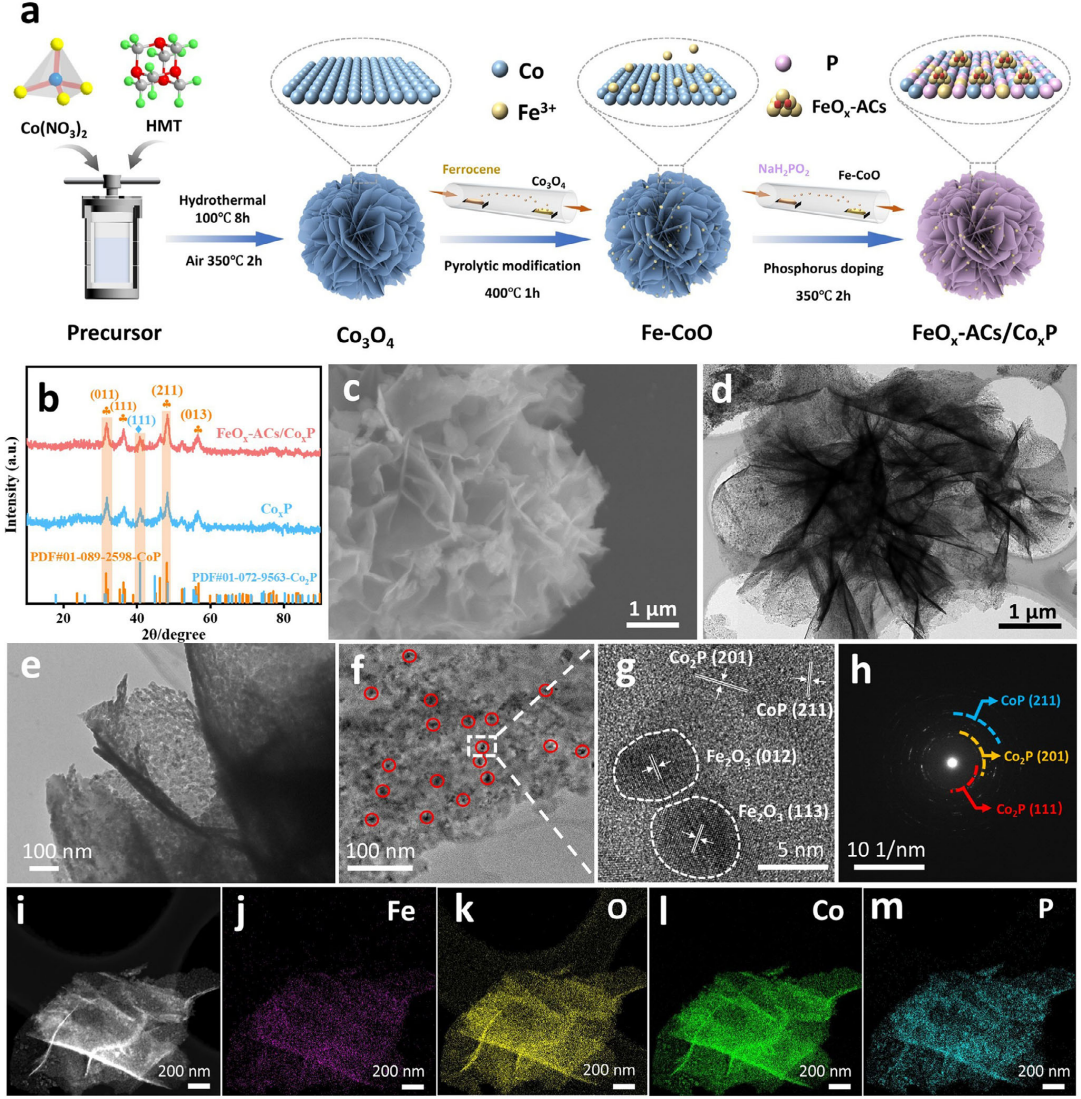
Synthesis and characterization of FeO_x_‐ACs/Co_x_P. a) Schematic of the synthesis of FeO_x_‐ACs/Co_x_P catalyst. b) XRD spectra of FeO_x_‐ACs/Co_x_P. c) SEM and d) TEM images of FeO_x_‐ACs/Co_x_P. e,f) Zoom in TEM images and g) HR‐TEM images of FeO_x_‐ACs/Co_x_P. h) SEAD pattern of FeO_x_‐ACs/Co_x_P. i–m) Corresponding elemental mapping of FeO_x_‐ACs/Co_x_P.

As illustrated in the schematic synthesis route (**Figure** **1**a), cobalt hydroxide (Co(OH)_2_) was initially synthesized as the precursor of cobalt through a hydrothermal strategy, in which Hexamethylenetetramine (HMT) as alkaline source gradual decomposed to generate NH_3_, enabling a controlled, slow elevation of the OH^−^ concentration that facilitated anisotropic crystal growth. Then, the Co_3_O_4_ nanoflowers precursor was obtained through a single calcination process (Scanning electron microscope (SEM) and X‐ray diffraction (XRD) spectrum in Figure S1, Supporting Information). Subsequent iron incorporation was achieved through a CVD process, where ferrocene (Fe source) and cobalt hydroxide were positioned in the upstream and downstream zones, respectively. The resulted Fe‐CoO intermediate (XRD spectrum in Figure S2 (Supporting Information), SEM and Transmission electron microscopy (TEM) images in Figures S3 and S4 (Supporting Information) was then subjected to phosphorization using sodium hypophosphite as phosphorus source under inert atmosphere, ultimately forming Fe atomic clusters anchored on the surface of Fe doped CoP/Co_2_P nanosheets (FeO_x_‐ACs/Co_x_P). Meanwhile, we also synthesized Co_x_P (without Fe incorporation) as counter samples. XRD patterns of FeO_x_‐ACs/Co_x_P and Co_x_P in Figure 1b displayed similar peak positions, with the dominant characteristic peaks at 31.6°, 36.3°, and 48.1° corresponding to (011), (111), and (211) planes of CoP phase (JCPDS 01‐089‐2598), while the 40.8° peak matched (111) plane of Co_2_P (JCPDS 01‐072‐9563). Notably, no additional Fe‐based peak was detected in XRD patterns for FeO_x_‐ACs/Co_x_P, suggesting Fe doped into the crystal lattice of Co_x_P or forming tiny sized Fe atomic clusters. Inductively Coupled Plasma Optical Emission Spectrometry (ICP‐OES) analysis reveals an ultralow iron content of 2.4 wt.% with cobalt content of 39.7 wt.%. Besides, the Fourier Transform Infrared Spectroscopy (FTIR) spectrum of FeO_x_‐ACs/Co_x_P exhibits a characteristic Co‐P vibration band at 1260 cm^−1^, further confirming the successful preparation of Co_x_P (Figure S5, Supporting Information). SEM image in Figure 1c reveals that Fe‐ACs/Co_x_P well preserves the original 3D nanoflower configuration, demonstrating remarkable structural integrity without observable agglomeration or collapse during Fe incorporation and phosphorization processes. TEM images in Figures 1d and S6 (Supporting Information) further show the interconnected 2D nanosheet network forming open 3D frameworks. The zoomed‐in TEM images in Figure 1e reveals the porous structure and ultrathin nature of the FeO_x_‐ACs/Co_x_P composite. The above merits effectively enhance surface area and active sites exposure while maintaining structural stability. TEM images in Figure 1f and High‐resolution TEM (HR‐TEM) image (Figure 1g) clearly reveals the uniform distribution of Fe_2_O_3_ clusters with an average size of ≈5 nm on the nanosheet surface. Moreover, Figure S7 (Supporting Information) reveals distinct lattice fringes of 0.220, 0.203, and 0.189 nm, consistent with d‐spacing values of Co_2_P (111), Co_2_P (201), and CoP (211) planes. The corresponding selected‐area electron diffraction (SAED) pattern (Figure 1h) exhibits concentric diffraction rings also matching crystalline phases of Co_2_P and CoP. Elemental mapping through High‐Angle Annular Dark Field Scanning Transmission Electron Microscopy (HAADF‐STEM) (Figure 1i–m) confirms the homogeneous spatial distribution of Fe, O, Co, and P elements throughout the nanosheets. The SEM and TEM images of Co_x_P in Figures S8–S10 (Supporting Information) show a similar 3D flower‐like structure to FeO_x_‐ACs/Co_x_P. The lattice fringes, elemental mapping, and characteristic peaks confirmed the successful synthesis of Co_x_P, with homogeneous elemental distribution observed throughout. Brunauer–Emmett–Teller (BET) measurements, demonstrating that Fe incorporation and interfacial cluster construction lead to an increase in specific surface area (60.93 m^2^ g^−1^) compared to the Fe‐CoO (49.42 m^2^ g^−1^) and Co_x_P (22.12 m^2^ g^−1^) (Figure S11, Supporting Information).

**Figure 2:**
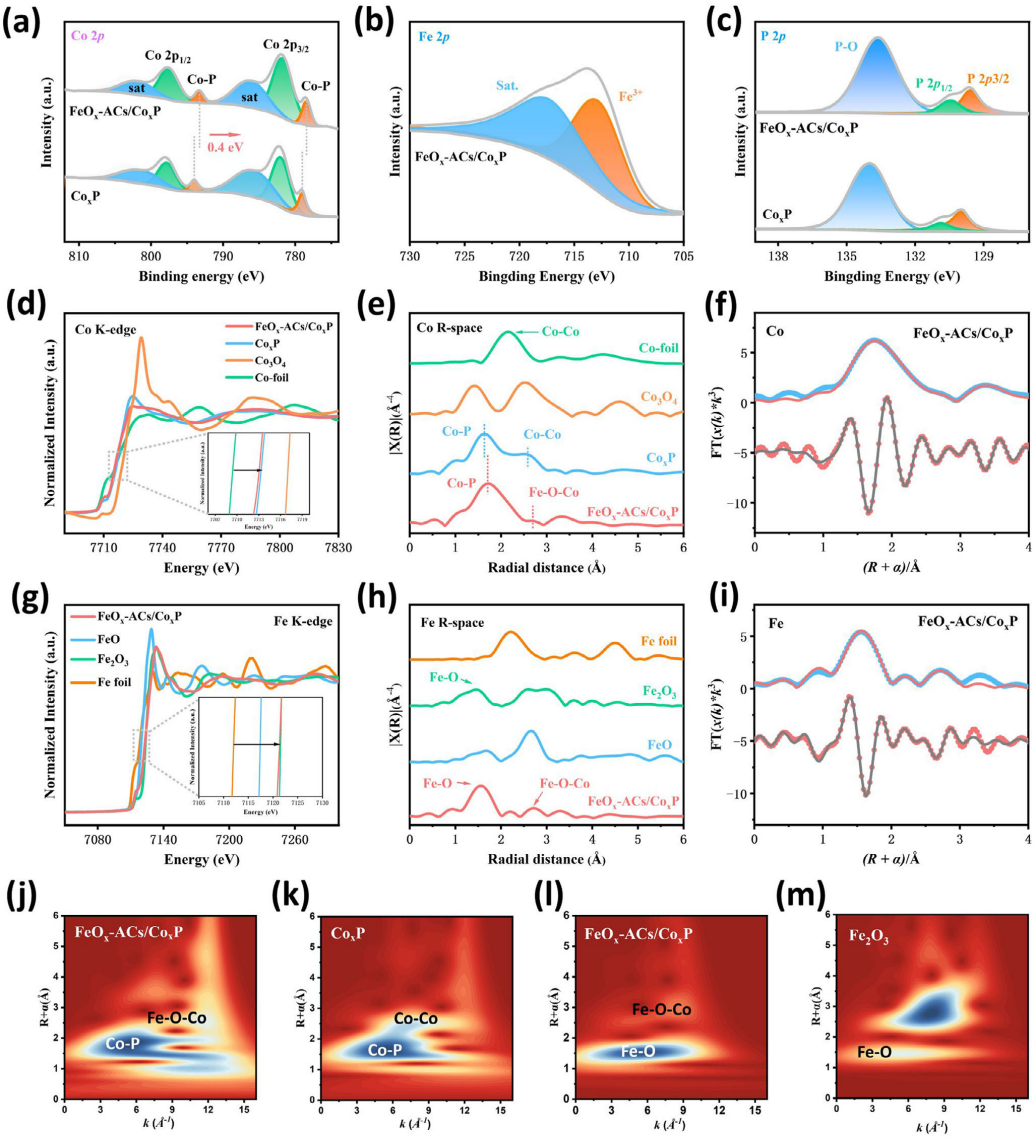
Electronic structure characterization of the catalyst. High‐resolution XPS spectra of a) Co 2p, b) Fe 2p, and c) P 2p. d) Co K‐edge XANES spectra of FeO_x_‐ACs/Co_x_P and control samples. e) Fourier‐transformed Co K‐edge EXAFS of FeO_x_‐ACs/Co_x_P and control samples. f) Co‐K edge FT‐EXAFS fitting curves of FeO_x_‐ACs/Co_x_P. g) Fe K‐edge XANES spectra of FeO_x_‐ACs/Co_x_P and control samples. h) Fourier‐transformed Fe K‐edge EXAFS of FeO_x_‐ACs/Co_x_P and control samples. i) Fe‐K edge FT‐EXAFS fitting curves of FeO_x_‐ACs/Co_x_P. j,k) WT‐EXAFS of FeO_x_‐ACs/Co_x_P and Co_x_P at the Co K‐edge. l,m) WT‐EXAFS of FeO_x_‐ACs/Co_x_P and Fe_2_O_3_ at the Fe K‐edge.

The electronic states and chemical composition of FeO_x_‐ACs/Co_x_P, Fe‐CoO, and Co_x_P were systematically characterized through X‐ray photoelectron spectroscopy (XPS). The XPS full survey spectrum of FeO_x_‐ACs/Co_x_P in Figure S12 (Supporting Information) shows the distinct characteristic peaks corresponding to Fe, O, Co, and P elements, aligning well with the elemental mapping and ICP‐OES observations (Table S1, Supporting Information). In the high‐resolution of Co 2p spectra of FeO_x_‐ACs/Co_x_P (**Figure** **2**a), the peaks located at 782.0 and 797.8 eV, accompanied by their satellite features at 786.5 and 802.4 eV, can be attributed to Co 2p_3/2_ and Co 2p_1/2_. The characteristic peaks at 778.0 and 792.9 eV were attributed to Co─P bonding.^[^ ^36^, ^37^, ^38^ ^]^ However, a negative shift of 0.4 eV in the Co─P binding energy was observed in FeO_x_‐ACs/Co_x_P compared to Co_x_P (777.6 and 792.5 eV), suggesting modified electronic environments around Co centers due to the presence of Fe atomic clusters. The XPS spectrum of the Fe 2p (Figure 2b) shows a typical Fe^3+^ characteristic peak (Fe 2p_3/2_) at 713.3 eV,^[^ ^39^, ^40^ ^]^ which may be attributed to the doped Fe species and oxidized Fe atomic clusters. The high valence state of Fe and negative shift of Co peaks suggest the electron redistribution occurs between Fe and Co species, where Fe acts as an electron donator and Co atoms as an electron acceptor, resulting in increased electron density of the coordinated Co atoms. The O 1s spectrum of FeO_x_‐ACs/Co_x_P (Figure S13, Supporting Information) shows three peaks at 529.2 eV (lattice oxygen bonded to Fe (O_L_)), 530.7 eV (hydroxyl groups), and 532.0 eV (adsorbed oxygen, O_ads_),^[^ ^41^ ^]^ The P 2p spectrum of FeO_x_‐ACs/Co_x_P (Figure 2c) shows two characteristic peaks at 129.7 and 130.5 eV, corresponding to the P 2p_3/2_ and P 2p_1/2_ states in metal phosphide species, respectively. The peak observed at 134.7 eV can be attributed to the P─O bond in surface metaphosphate species, which results from air oxidation,^[^ ^42^ ^]^ It is worth noting that the P─O bond exhibits a 0.3 eV negative shift in FeO_x_‐ACs/Co_x_P relative to Co_x_P, which can be attributed to Fe doping‐induced electron transfer from the 3d orbital of Fe to the 3p orbital of P. This electron delocalization induces an elevation of electron density surrounding P atoms, which consequently weakens the binding effect of nuclear charge on the P atom in P─O bonds. We also conducted XPS characterization of the control sample Fe‐CoO, which in agreement with the expected chemical state and bonding environment (Figure S14, Supporting Information).

Systematic X‐ray absorption spectroscopy (XAS) investigations were conducted to further elucidate the oxidation states and local coordination environments of constituent elements of FeO_x_‐ACs/Co_x_P. The Co K‐edge X‐ray absorption near‐edge structure (XANES) spectra in Figure 2d reveals an absorption edge energy downshift in FeO_x_‐ACs/Co_x_P compared to Co_x_P, indicating a lower Co oxidation state of FeO_x_‐ACs/Co_x_P. The Fourier transformed (FT) k^3^‐weighted extended X‐ray absorption fine structure (FT‐EXAFS) spectra of Co for FeO_x_‐ACs/Co_x_P reveal two dominant peaks, which are ascribed to Co─P and Co─Co coordination, respectively (Figure 2e,f).^[^ ^43^ ^]^ It is noteworthy that the incorporation of Fe species induces extended Co─P bond length in FeO_x_‐ACs/Co_x_P compared to pristine Co_x_P. EXAFS fitting results further reveal a decrease in the coordination number N(Co─P) from 5.8 in Co_x_P to 3.2 in FeO_x_‐ACs/Co_x_P, indicating a weakening of Co─P coordination (Figure S15, Tables S2–S4, Supporting Information).^[^ ^44^ ^]^ This change may arise from lattice expansion induced by Fe substitution creating asymmetric coordination environments or the interaction of Fe atomic clusters with surface Co─P unit. Concurrently, the weakened Co─Co coordination peak intensity confirms reduced Co─Co bonds, highlighting the critical role of Fe incorporation in manipulating coordination configurations. The extended Co─P bond length may alter the electronic environment of the cobalt center by reducing electron density delocalization from P to Co, and also facilitate the in situ transformation into the active Co oxyhydroxide phase during OER, thereby enhancing catalytic activity. Fe K‐edge absorption edge of FeO_x_‐ACs/Co_x_P (Figure 2g) is higher than FeO and closely to Fe_2_O_3_, indicating an average oxidized state for Fe species, which is consistent with the Fe 2p orbital features observed by XPS. The FT‐EXAFS spectra of Fe for FeO_x_‐ACs/Co_x_P and its fitting curve (Figure 2h,i) reveal two distinct coordination peaks in FeO_x_‐ACs/Co_x_P (Fe─O and Fe─Fe/Co). The presence of Fe─O coordination bonds confirms the formation of oxidized Fe species.^[^ ^45^ ^]^ Additionally, the Fe─Fe/Co bond exhibits a 0.17 Å shift compared to pure Fe foil, attributing to decreased electron density caused by Fe to Co charge transfer (Tables S5 and S6Supporting Information). The Fe‐O‐Co path further reveals interactions between Fe and Co atoms, representing a second‐shell coordination feature.^[^ ^46^, ^47^ ^]^ These observations collectively demonstrate that Fe not only doped to the crystal structure of Co_x_P, but also forming heterogeneous Fe_2_O_3_ nanoclusters on the surface.^[^ ^48^, ^49^ ^]^ The fitting data of other related samples further prove the successful synthesis of the material (Figure S16, Supporting Information). These coordination environment variations were further corroborated by wavelet transform (WT) EXAFS results (Figures 2j–m, S17, Supporting Information). This dual metallic center configuration facilitates synergistic electronic effects with the Fe‐O‐Co bridge enhancing charge transfer through electron delocalization.

### 2.1 Electrochemical Performance

**Figure 3:**
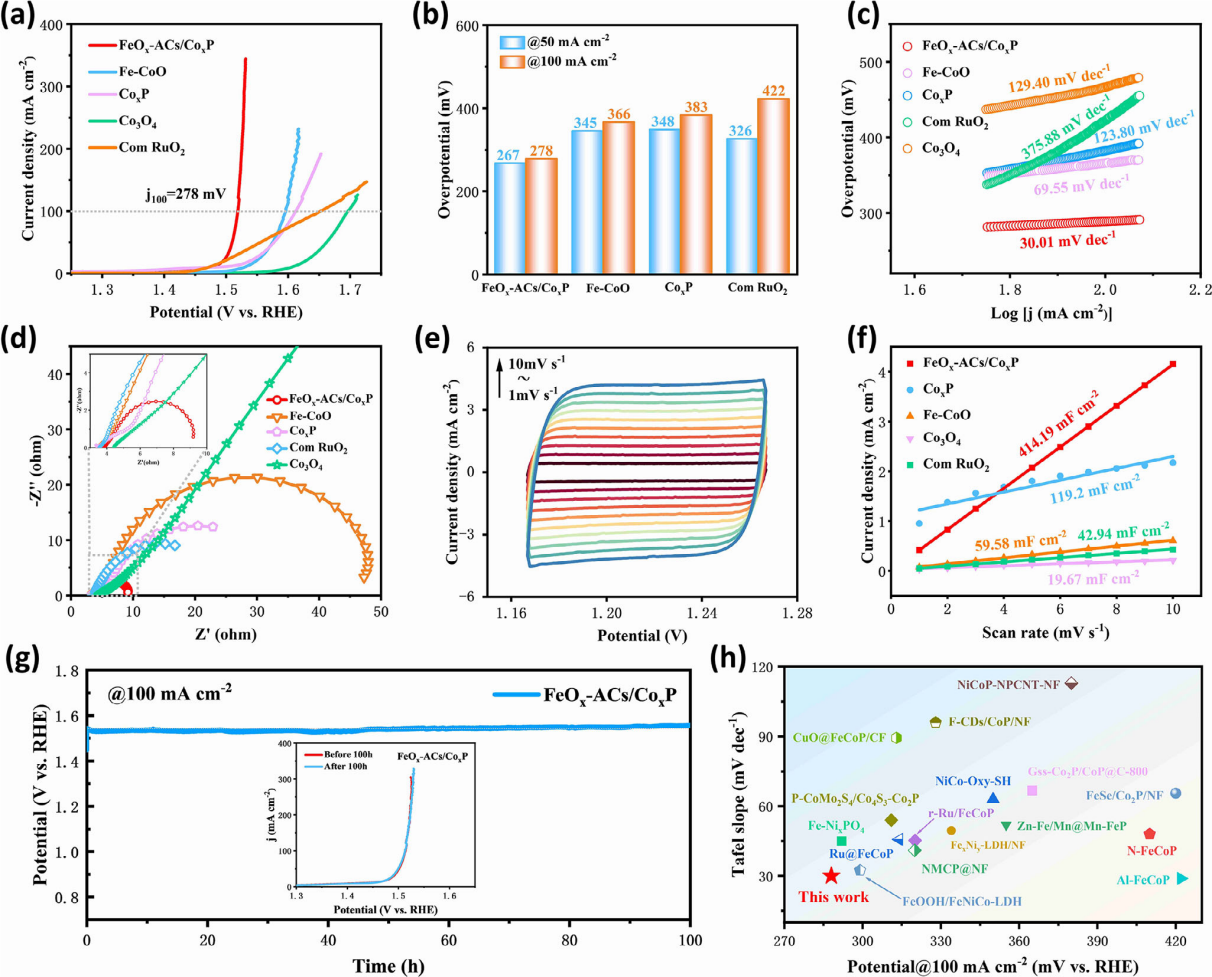
Electrochemical performance of FeO_x_‐ACs/Co_x_P in 1.0 M KOH. a) Polarization curves for OER activity of FeO_x_‐ACs/Co_x_P, Fe‐CoO, Co_x_P, Co_3_O_4,_ and Commercial RuO_2_ samples. b) Corresponding overpotentials at 50 and 100 mA cm^−2^ of FeO_x_‐ACs/Co_x_P and control samples for OER, respectively. c) Tafel slopes of FeO_x_‐ACs/Co_x_P and control samples for OER. d) Nyquist plots of FeO_x_‐ACs/Co_x_P and control samples. e) CV curves of the FeO_x_‐ACs/Co_x_P recorded at different scan rates. f) Electrochemical double layer capacitances of FeO_x_‐ACs/Co_x_P and control samples. g) Chronopotentiometric measurements of FeO_x_‐ACs/Co_x_P and control samples at 100 mA cm^−2^. h) Comparison of the voltage and stability time over FeO_x_‐ACs/Co_x_P and other reported catalysts at 100 mA cm^−2^ for OER.

The OER catalytic performance of the synthesized materials was systematically assessed using a conventional three‐electrode configuration in 1.0 M KOH electrolyte. We systematically investigated the effects of varying Fe precursor amounts and phosphidation ratios. The optimal electrochemical performance was achieved at a Co mass feeding ratio of 6:1 and a phosphidation ratio of 10:1 (Figure S18, Supporting Information). As the results shown in **Figure** **3**a,b, FeO_x_‐ACs/Co_x_P exhibits superior OER catalytic activity, requiring 278 mV overpotential to achieve a current density of 100 mA cm^−2^ with an exceptionally low Tafel slope of 30.01 mV dec^−1^ (Figure 3c). This performance substantially outperforms control catalysts including Co_x_P (η_100_ = 383 mV, 123.8 mV dec^−1^), Fe‐CoO (η_100_ = 366 mV, 69.55 mV dec^−1^), and commercial RuO_2_ (η_100_ = 422 mV, 375.88 mV dec^−1^). The decreased overpotential and Tafel slope indicate enhanced catalytic efficiency and accelerated reaction kinetics of FeO_x_‐ACs/Co_x_P. Remarkably, under high current conditions (Figure S19, Supporting Information), FeO_x_‐ACs/Co_x_P achieves an ultra‐high current density of 700 mA cm^−2^ with merely 300 mV overpotential, highlighting its exceptional catalytic capability. Electrochemical impedance spectroscopy (EIS) results (Figure 3d) reveal the lowest charge transfer resistance (R_ct_ = 5.4 Ω) for FeO_x_‐ACs/Co_x_P among all tested catalysts, confirming improved charge transfer kinetics during OER processes. The enhanced electrochemical performance was further supported by cyclic voltammetry (CV) measurements (Figures 3e and S20, Supporting Information). FeO_x_‐ACs/Co_x_P demonstrates a double layer capacitance (*C*_dl_) of 414.19 mF cm^−2^, representing multiplication compared to Co_x_P (119.2 mF cm^−2^) and commercial RuO_2_ (42.94 mF cm^−2^), respectively (Figure 3f). This capacitance enhancement suggests Fe incorporation induces favorable electronic structure modifications that promote surface reconstruction and expose additional active sites. Long‐term stability evaluation through chronopotentiometry (CP) testing (Figure 3g) shows excellent durability with 98.7% potential retention after 100 h of operation at 100 mA cm^−2^ in alkaline electrolyte. The outstanding activity and stability position the 3D nanoflower‐structured FeO_x_‐ACs/Co_x_P as a superior alternative to most reported OER materials (Figure 3h; Table S7, Supporting Information), with its structural advantages in facilitating charge transfer and active site accessibility being key to the observed performance enhancements.

### 2.2 Mechanism Analysis of Enhanced OER Activity

**Figure 4:**
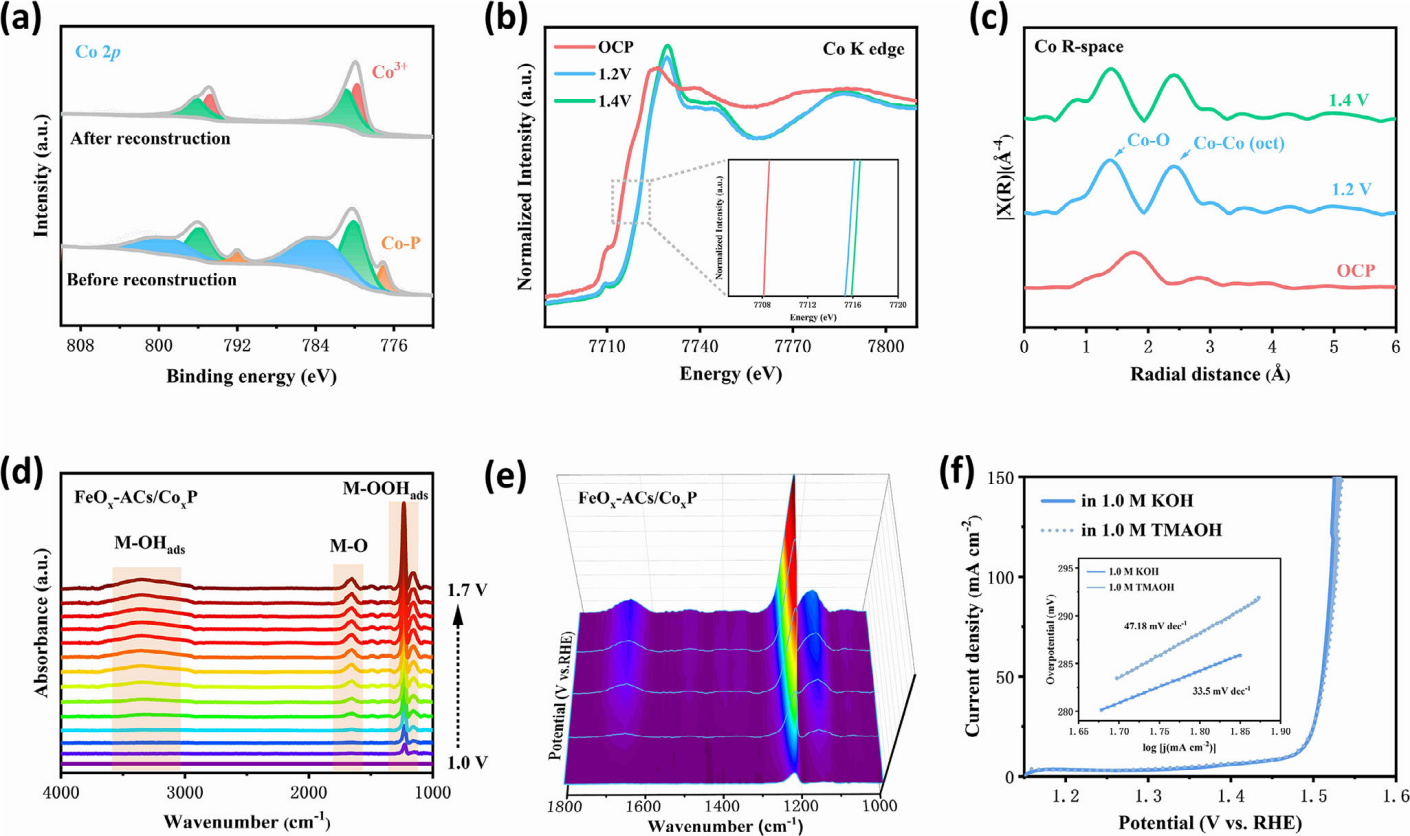
a) The Co 2p spectrums of FeO_x_‐ACs/Co_x_P before and after the OER long term stability test. b) *Operando* Co K‐edge XANES spectra of FeO_x_‐ACs/Co_x_P during the OER process with applied potentials from OCP to 1.6 V. Inset: Oxidation state changes of Co in FeO_x_‐ACs/Co_x_P. c) *Operando* Co K‐edge EXFAS spectra of FeO_x_‐ACs/Co_x_P. d,e) *Operando* ATR‐SEIRAS FTIR spectroscopic investigation of FeO_x_‐ACs/Co_x_P. f) LSV curves of FeO_x_‐ACs/Co_x_P in 1.0 M KOH and 1.0 M TMAOH, respectively.

FeO_x_‐ACs/Co_x_P has demonstrated excellent OER catalytic activity and stability, which may be attributed to the unique atomic structure and synergistic charge redistribution effect through iron incorporation and cluster structure modification. The XPS after long‐term stability tests have been conducted to investigate the surface reconstruction and intrinsic catalytic sites during OER process. The Co 2p spectra results in **Figures** **4**a, S21a and S22a (Supporting Information) shows that the Co─P bonds in FeO_x_‐ACs/Co_x_P and Co_x_P both disappeared and transformed into Co─O bonds. This transformation results from the chemisorption reaction between surface phosphides and water, generating OH^−^ species (CoOOH), while Co on the surface gradually oxidized to higher valence states (Co^3+^). The Fe 2p spectra show a higher content of Fe^3+^ compared to the sample before the long‐term stability (Figure S21b, Supporting Information). The O 1s spectra showed increased metal‐O content (Figure S21c, Supporting Information), indicating the formation of newly evolved CoO_x_, CoOOH, and FeOOH species on the catalyst surface, confirming the dynamic reconstruction process of the phosphide catalysts in electrolyte. The peak at 535.2 eV corresponds to O‐F_x_ species originating from the Nafion solution added during catalyst ink preparation. Meanwhile, the conversion of Co─P bonds to P─O bonds on both FeO_x_‐ACs/Co_x_P and Co_x_P surfaces in P 2p spectra suggests possible phosphate formation (Figure S21d, Supporting Information), which facilitates water molecule adsorption and maintains the dynamic equilibrium at the reaction interface,^[^ ^50^ ^]^ However, after prolonged stability testing, the P signal nearly disappeared in the Co_x_P sample (Figure S22b, Supporting Information), indicating its poor stability with P species being easily lost during extended OER operation. To further understand the origin of catalytic performance of FeO_x_‐ACs/Co_x_P, we conducted *Operando* spectroscopic characterization to investigate the OER catalytic mechanism. As shown in Figure 4b, the in situ Co K‐edge XANES spectra reveal a gradual positive shift in the absorption edge with increasing applied potential, signifying an increase of the Co oxidation state. FT curves of the Co K‐edge EXAFS spectra (Figure 4c) demonstrate that the Co species in FeO_x_‐ACs/Co_x_P component reconstructs to a highly active phase CoOOH beyond 1.2 V vs RHE. *Operando* Attenuated Total Reflection Surface‐Enhanced Infrared Absorption Spectroscopy (*Operando* ATR‐SEIRAS) analysis was systematically performed on Fe‐ACs/Co_x_P and Co_x_P under alkaline conditions to identify reaction intermediates and evaluate spatial interaction effects during the OER process. There are three characteristic infrared vibrational features observed at ≈3300 cm^−1^, ≈1650 cm^−1^, and 1250‐1120 cm^−1^ for FeO_x_‐ACs/Co_x_P and Co_x_P, which are assigned to O_ads_, hydroxyl groups and superoxide intermediates (OOH_ads_), respectively,^[^ ^51^, ^52^, ^53^ ^]^ These three potential‐dependent vibrational bands that intensify with increasing applied potential, suggesting both FeO_x_‐ACs/Co_x_P and Co_x_P follow the adsorbate evolution mechanism (AEM) pathway during OER process (Figure 4d,e). Notably, the characteristic peak at ≈1650 cm^−1^ emerges at 1.15 V for FeO_x_‐ACs/Co_x_P, which is earlier than that of Co_x_P (≈1.35 V) (Figure S23, Supporting Information). This shift indicates reduced adsorption energy of *OOH intermediates and facilitates earlier formation of M‐O active sites, consequently improving intermediate conversion efficiency and OER kinetics. Moreover, tetramethylammonium cations (TMA^+^) were employed as chemical probes to further investigate the OER catalytic mechanism due to their strong electrostatic interactions with oxygen intermediates (*O_2_^2−^ or *O_2_^−^ generated in lattice oxygen mechanism (LOM) pathway),^[^ ^54^, ^55^ ^]^ As the LSV results shown in Figures 4f and S24 (Supporting Information), FeO_x_‐ACs/Co_x_P nanosheets maintain comparable OER activity in 1.0 M TMAOH electrolyte vs 1.0 M KOH, and almost no decrease in activity has been observed, as compared to the control samples, which indicates no generation of negatively charged oxygen species during OER. This observation further demonstrates FeO_x_‐ACs/Co_x_P follow the AEM pathway.

### 2.3 DFT Calculations

**Figure 5:**
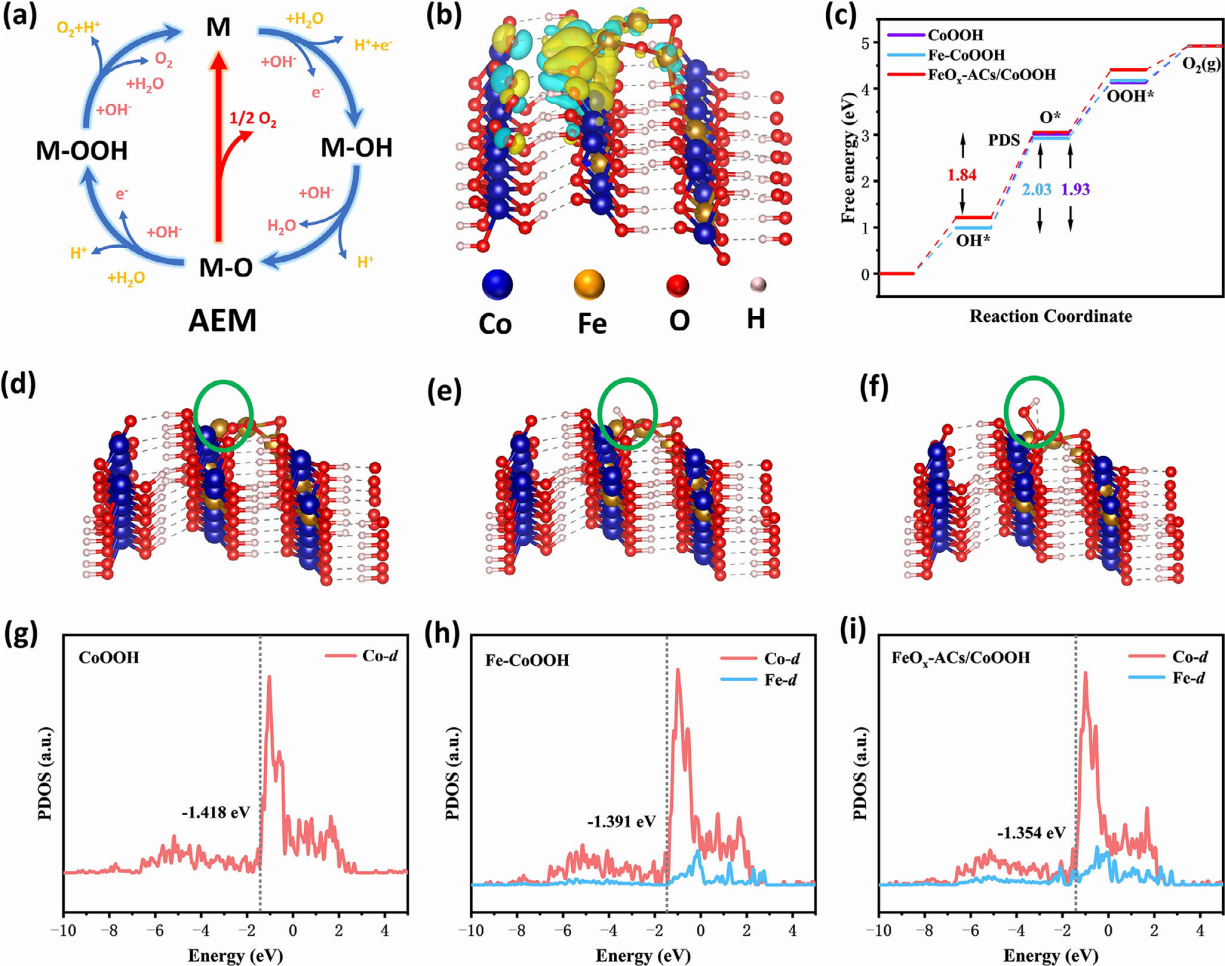
a) The schematic illustration of the AEM pathway. b) The charge density difference of the FeO_x_‐ACs/CoOOH (the yellow and light blue areas represent accumulation and charge depletion, respectively). Color code: blue denotes Co, orange denotes Fe, red denotes O, and white denotes H. c) Calculated Gibbs free energy diagram of OER following AEM on the surface of different catalysis. d–f) FeO_x_‐ACs/CoOOH structure model. The PDOS for g) CoOOH and h) Fe‐CoOOH. i) FeO_x_‐ACs/CoOOH.

Density functional theory (DFT) calculations were conducted to further investigate the active sites and reaction mechanism of FeO_x_‐ACs/Co_x_P for OER. Based on the AEM (**Figure** **5**a) identified in previous mechanistic studies of OER, and guided by the structural evolution to CoOOH observed in *operando* XAS (Figure 4b,c) as well as the surface composition analysis from pre‐/post‐stability XPS, we attribute the high OER activity of FeO_x_‐ACs/Co_x_P in alkaline media to its in situ transformation into the highly active CoOOH phase based on the post XPS and in *operando* XAS results (Figure 4a,b), Therefore, we have chosen hydroxide phase as model for the DFT calculation. We constructed a FeO_x_‐cluster stabilized on Fe doped CoOOH model was constructed to represent FeO_x_‐ACs/Co_x_P to investigate the origin of OER activity (named as FeO_x_‐ACs/CoOOH). For comparison, two additional models were established: Fe‐doped CoOOH (named as Fe‐CoOOH) and pristine CoOOH (named as CoOOH). Charge density difference analysis in Figure 5b revealed electron transfer from FeO_x_ clusters to CoOOH through Fe‐O‐Co bridges, inducing spatial electron redistribution on Fe and Co species, consistent with XAS and XPS results. The free energies of OER intermediates (*OH, *O, *OOH) were evaluated, with corresponding adsorption models illustrated in Figures 5c–f and S25 (Supporting Information). As depicted in Figure 5c, the Gibbs free energy change (ΔG) for the conversion of *OH to *O for the three models reaches its maximum value among the distinct steps involving *OH, *O, *OOH, and O_2_. This indicates that *O formation constitutes the RDS. The calculated ΔG for RDS was 1.84 eV on FeO_x_‐ACs/CoOOH, 1.93 eV on Fe‐CoOOH, and 2.03 eV on CoOOH, demonstrating Fe doping and surface FeO_x_ cluster formation are capable of effectively reducing the Gibbs free energy barrier and accelerating the catalytic kinetics.^[^ ^56^ ^]^ Furthermore, the density of states (DOS) at the Fermi level was higher for FeO_x_‐ACs/CoOOH than pristine CoOOH and Fe‐CoOOH (Figure S26, Supporting Information), indicating enhanced electron supply during catalysis. The d‐band center position (ε_d_) is a key descriptor governing the adsorption strength of reaction intermediates (*e.g.*, *O, *OH, *OOH) on transition metal catalyst surfaces, directly correlating with catalytic adsorption capacity,^[^ ^57^ ^]^ Projected DOS (PDOS) analysis showed that the d‐band center of FeO_x_‐ACs/CoOOH (−1.354 eV) was closer to the Fermi level compared to CoOOH (−1.418 eV) and Fe‐CoOOH (−1.391 eV) (Figure 5g–i). This shift indicates that Fe doping induces electronic restructuring at the metal active sites, enhancing their adsorption capability toward oxygen‐containing intermediates. Furthermore, the formation of FeO_x_ nanoclusters optimizes the local coordination environment of these active sites, promoting more stable adsorption of intermediates on the catalyst surface. These modifications collectively facilitate charge delocalization and improve electrical conductivity,^[^ ^58^, ^59^ ^]^ This facilitates efficient electron transfer between Fe/Co sites and intermediates, enhancing OER kinetics.

### 2.4 OER Performance in Alkaline Seawater and AEM‐WE Devices

**Figure 6:**
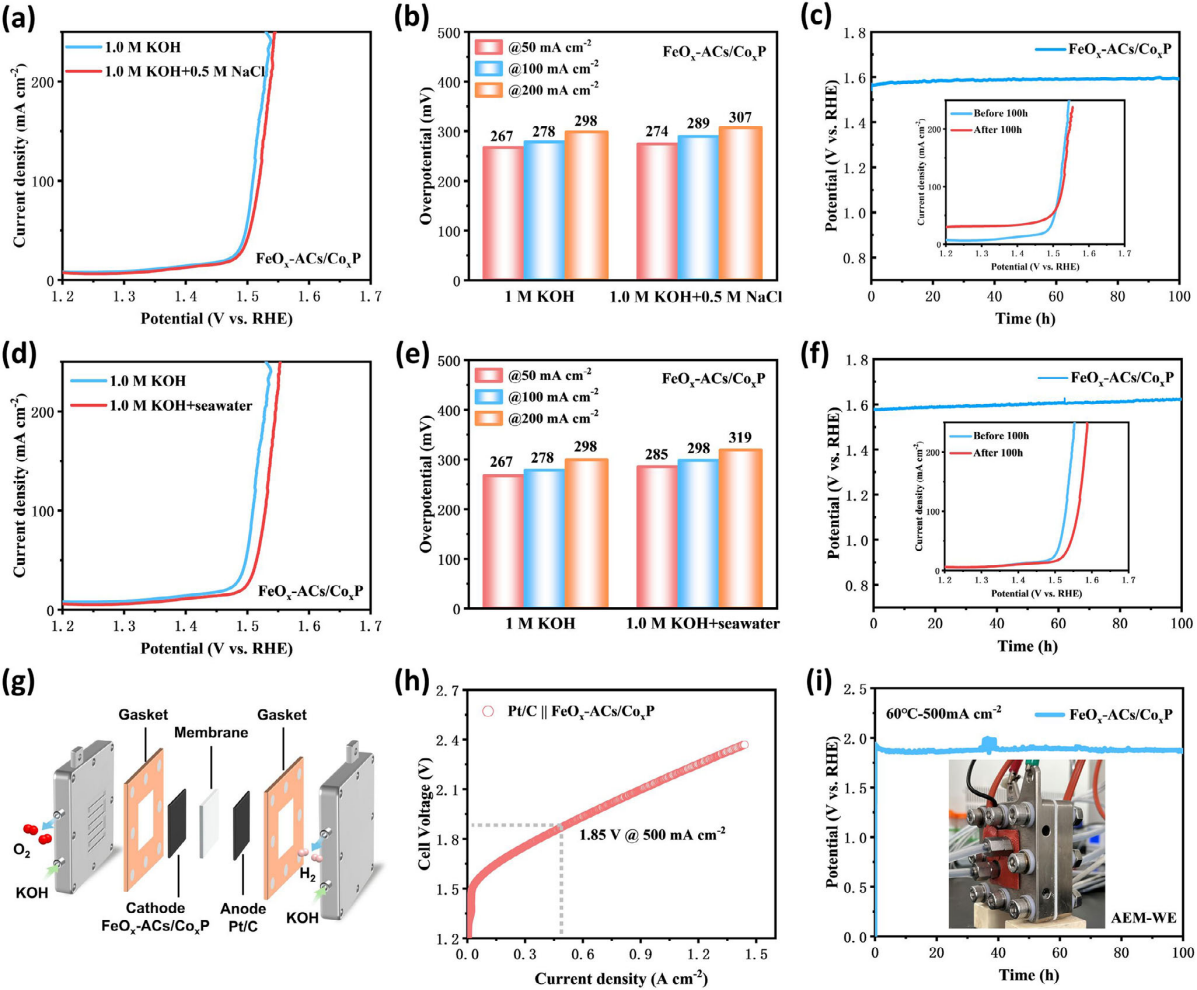
a) LSV plots of FeO_x_‐ACs/Co_x_P in 1.0 M KOH and 1.0 M KOH + 0.5 M NaCl. b) the comparison of overpotential at different current densities in 1.0 M KOH and 1.0 M KOH + 0.5 M NaCl, and c) Durability test at 100 mA cm^−2^. d) LSV plots of FeO_x_‐ACs/Co_x_P in 1.0 M KOH and 1.0 M KOH + seawater. e) the comparison of overpotential at different current densities in 1.0 M KOH and 1.0 M KOH + seawater, and f) Durability test at 100 mA cm^−2^. g) Schematic diagram of AEM‐WE electrolyzer composed of cathodic FeO_x_‐ACs/Co_x_P and anodic Pt/C. h) Steady‐state polarization curves of Pt/C || FeO_x_‐ACs/Co_x_P electrolyzer. i) Long‐term stability test of AEM‐WE with FeO_x_‐ACs/Co_x_P as the cathode and Pt/C as the anode at 500 mA cm^−2^ and 60 °C. Inset: The actual operation figure of the AEM‐WE electrolyzer.

To evaluate the performance of the FeO_x_‐ACs/Co_x_P catalyst under practical conditions, its OER activity and chloride tolerance were systematically investigated in the following alkaline electrolytes: 1.0 M KOH + 0.5 M NaCl and 1.0 M KOH + natural seawater. The Linear sweep voltammetry (LSV) results in **Figure** **6**a,b reveals that FeO_x_‐ACs/Co_x_P maintain excellent OER activity in the alkaline electrolyte containing 0.5 M NaCl, comparable to that in pure 1.0 M KOH. In natural seawater, OER activity slightly decreased due to mild electrode poisoning caused by contaminants such as microorganisms, Mg^2+^/Ca^2+^ ions, and suspended particles,^[^ ^60^, ^61^ ^]^ Notably, FeO_x_‐ACs/Co_x_P still exhibits outstanding electrocatalytic performance in alkaline seawater, achieving overpotentials of 298 mV to reach a current density of 100 mA cm^−2^ (Figure 6d,e). Furthermore, FeO_x_‐ACs/Co_x_P exhibits excellent stability under a constant current density of 100 mA cm^−2^ for 100 h, showing only a slight potential decay of 8 mV in 1.0 M KOH + 0.5 M NaCl and 32 mV in 1.0 M KOH + seawater (Figure 6c,f). The excellent activity and stability make FeO_x_‐ACs/Co_x_P an outstanding alternative to the reported OER materials for alkaline seawater (Figure S27, Table S8, Supporting Information). These results demonstrate the exceptional performance of FeO_x_‐ACs/Co_x_P in both simulated and real seawater environments. We further constructed an anion‐exchange membrane water electrolysis (AEM‐WE) device using FeO_x_‐ACs/Co_x_P as the anode and Pt/C as the cathode to assess the practical application (Figure 6g). Under 60 °C, the device requires only 1.85 V to achieve a current density of 500 mA cm^−2^ and demonstrated stable operation at 500 mA cm^−2^ for 100 h (Figure 6h,i), exhibiting outstanding stability and promising practical application potential.

## 3. Conclusion

In this study, we precisely controlled the formation of surface clusters using the CVD method and synthesized FeO_x_‐ACs/Co_x_P as an efficient electrocatalyst for alkaline water and seawater electrolysis. The catalyst achieves low overpotentials of 278 mV (1.0 M KOH) and 298 mV (1.0 M + natural alkaline seawater) at a current density of 100 mA cm^−2^, while maintaining stability for over 100 h. The constructed Fe‐O‐Co interfacial bridges optimize electron density distribution by serving as electron donors to modulate the electronic density and coordination environment around cobalt centers, concurrently elongating Co─P bonds. In situ characterization and DFT calculations reveal the dynamic structural evolution of high‐valence Co active center, which modulates the adsorption free energy of *OOH intermediates via an AEM mechanism, thereby lowering the reaction energy barrier during the OER. The AEW‐WE device incorporating FeO_x_‐ACs/Co_x_P as the anode catalyst and Pt/C as the cathode demonstrates excellent performance, achieving a cell voltage of only 1.85 V at a current density of 500 mA cm^−2^. This work highlights the advantages of a precise nanocluster engineering strategy for developing efficient OER catalysts for practical applications.

## Conflict of Interest

The authors declare the following financial interests/personal relationships which may be considered as potential competing interests: Hao Liu reports financial support was provided by Australian Research Council. If there are other authors, they declare that they have no known competing financial interests or personal relationships that could have appeared to influence the work reported in this paper.

## Supporting information



Supporting Information

## Data Availability

Research data are not shared.
