# Supplementary material for: Modulating Local Electronic Structure via Cluster Engineering on Cobalt Phosphide for Efficient Water/Seawater Splitting
Source: Adv Sci (Weinh). 2026 Mar 24;13(23):e20390. doi: 10.1002/advs.202520390 (PMC13104125; doi:10.1002/advs.202520390)
Supplement: Supplementary file 1 — Supporting Information [file ADVS-13-e20390-s001.docx]

**Supporting Information**

**Modulating Local Electronic Structure via Cluster Engineering on Cobalt Phosphide for Efficient Water/Seawater Splitting**

*Cheng Gong^1,2^, Fengying Pan^1^, Pengpeng Zhang^1^, Yuhan Xie^3^, Yaojie Lei^2^, Xiaobo Zheng^2^, Dinushi Munasinghe* *Mudiyanselage^2^, Hong Gao^1^, Jinqiang Zhang^2,*^, Yufei Zhao^2,*^, Guoxiu Wang^2^, Hao Liu^2,*^*

^1^ Joint International Laboratory on Environmental and Energy Frontier Materials, School of Environmental and Chemical Engineering, Shanghai University, Shanghai 200444, China

^2^ School of Mathematical and Physical Sciences, Faculty of Science, University of Technology Sydney, Broadway

^3^ School of Chemistry, The University of New South Wales, Sydney, NSW, 2052, Australia

^*^ Corresponding authors.

E-mail addresses: jinqiang.zhang@uts.edu.au (J. Zhang), yufei.zhao@uts.edu.au (Y. Zhao), hao.liu@uts.edu.au (H. Liu).

Cheng Gong and Fengying Pan contributed equally to this work.

**Section S1 Experimental Section**

- 1. **Chemicals and Materials**

Cobalt (II) Nitrate Hexahydrate (Co(NO_3_)_2_·6H_2_O), Ruthenium (IV) Oxide (RuO_2_) and absolute ethanol were purchased from Shanghai Titan Chemical Co., Ltd., where Co(NO_3_)_2_·6H_2_O was of the brand Adamas-life^®^. Hexamethylenetetramine (HMT) was obtained from Sinopharm Chemical Reagents Co., Ltd. Sodium Hypophosphite Monohydrate (NaH_2_PO_2_·H_2_O) was purchased from Leyan, Shanghai, China (Product No. 1214830). Ferrocene (Fe(C_5_H_5_)_2_) was purchased from Shanghai Macklin Biochemical Technology Co., Ltd. All chemicals were used as received without further purification.

- 1. **Materials Preparation**

**Preparation of Co_3_O_4_**

Typically, Co_3_O_4_ nanosheets were synthesized by dissolving Co(NO_3_)_2_·6H_2_O (5 mmol) and HMT (10 mmol) in deionized water (30 mL) under 15-min ultrasonication, followed by 30-min magnetic stirring to achieve a homogeneous solution. The mixture was hydrothermally treated in a Teflon-lined autoclave at 100°C for 12 h. The resulting precipitate was repeatedly washed with deionized water/ethanol, vacuum-dried at 60°C for 12 h, and calcined in air at 400°C for 4 h to obtain Co_3_O_4_.

**Preparation of Fe-CoO**

The Fe-CoO composite was fabricated through a Chemical Vapor Deposition (CVD) strategy. In a typical synthesis, 25 mg as-prepared Co_3_O_4_ and Fe(C_5_H_5_)_2_ with different content (0, 75, 150, 225 and 300 mg) were put at two separate positions in a porcelain boat, with ferrocene at the upstream side. The system subsequently experienced a thermal treatment at 350°C for 2 h in 5% H_2_/Ar atmosphere with a heating rate of 5°C min^-1^.

**Preparation of Co_x_P**

The Co_x_P composite was fabricated through a CVD strategy. In a typical synthesis, 25 mg as-prepared Co_3_O_4_ and NaH_2_PO_2_·H_2_O with different content (0, 50, 125, 250 mg and 500 mg) were put at two separate positions in a porcelain boat, with NaH_2_PO_2_·H_2_O at the upstream side. The system subsequently experienced a thermal treatment at 400°C for 2 h in Ar atmosphere with a heating rate of 5°C min^-1^.

**Preparation of FeO_x_-ACs/Co_x_P**

Subsequently, the as-prepared Fe-CoO (25 mg) and NaH_2_PO_2_·H_2_O (250 mg) were similarly arranged in separate zones (NaH_2_PO_2_·H_2_O upstream) within a porcelain boat. A secondary annealing under pure Ar at 400°C for 2 h (heating rate: 5°C min^-1^) to form FeO_x_-ACs/Co_x_P.

**CVD synthesis process**

In our CVD synthesis, a tube furnace served as the reaction chamber housing both the iron precursor and substrate. Ferrocene, employed as the iron source, was positioned upstream within the furnace. Approximately 20 cm downstream from the precursor, the substrate material was placed. A carrier gas stream, controlled at a flow rate of 5 ~ 10 mL min^-1^, was introduced from the upstream end. During the heating process, the ferrocene precursor vaporizes upstream and is transported by the carrier gas to deposit onto the downstream substrate, thereby achieving the chemical vapor deposition.

- 1. **Material Characterizations**

Scanning electron microscope (SEM, JSM-7500F), transmission electron microscopy (TEM, JEM-F200) and high-angle annular dark-field scanning transmission electron microscopy (HAADF-STEM, JEM-ARM300F) were used to observe morphologies, structures and compositions of samples. X-ray diffraction (XRD) patterns were taken on a D/MAX2200V PC ((Cu Kα (λ= 0.15406 nm), 3 kV, 80 mA). Fourier Transform Infrared Spectroscopy (FTIR) were collected by Thermo Scientific Nicolet iS10. X-ray photoelectron spectroscopy (XPS) patterns were carried out on a Thermo Fisher Scientific K-Alpha instrument. Brunauer-Emmett-Teller (BET) Specific surface area measurements were collected by Micromeritics instrument (Shanghai) Ltd. (ASAP2460). Inductively Coupled Plasma Optical Emission Spectrometry (ICP-OES) was collected by Thermo Scientific iCAP 7200. Operando FTIR measurements were conducted on attenuated total reflection surface enhanced infrared absorption spectroscopy (ATR-SEIRAS) (Nicolet iS50). X-ray absorption spectra (XAS) were collected at the Australian National Synchrotron (Melbourne, Australia) and processed using ATHENA software.

- 1. **Electrochemical Measurements**

The OER performance was evaluated on a CHI 660E Electrochemical Workstation (CHI Instruments, Shanghai Chenhua Instrument Corp., China) with a standard three-electrode setup at room temperature. As-prepared catalysts were directly used as the working electrodes. The catalyst ink was prepared by dispersing the catalyst powder in a mixed solvent of ethanol and isopropanol (1:6, v/v), followed by the addition of 5 wt% Nafion solution (approximately 5 vol% relative to the total solvent volume). After thorough homogenization via ultrasonication, a uniform ink with a catalyst concentration of 5 mg mL^-1^ was obtained. This ink was then drop-cast onto Toray carbon paper (TGPH060) in multiple aliquots (0.25 cm^-2^). The catalyst loading on the carbon paper substrate was controlled at 2 mg cm^-2^ for all electrodes employed in this work. Hg/HgO electrode and Pt foil were used as the reference electrode and the counter electrode, respectively. The OER performance were evaluated in 1.0 M KOH solution at a scan rate of 5 mV s^-1^ by linear sweep voltammograms (LSV). In order to compensate for the effect of solution resistance, potentials were corrected by using the following equation: E_iR-corrected_ = E - iR*90%, in which R is the uncompensated ohmic resistance of the solution. Electrochemical impedance spectroscopy (EIS) tests were carried out at a scanning frequency ranging from 0.01 Hz to 100 kHz with an amplitude of 5 mV. The electrochemical double-layer capacitance (*C*_dl_) was estimated from typical cyclic voltammetry (CV) measurements with various scan rates (10, 20, 30, 40, 50, 60, 70, 80 90 and 100 mV s^-1^) in 0.05 ~ 0.15 V vs. Hg/HgO. The *C*_dl_ can be further converted into electrochemical active surface area (ECSA) using the specific capacitance value for a standard with 1.0 cm^-2^ real surface area. ECSA is a key factor quantifying the intrinsic catalytic activity of catalysts. The calculation equation is as follows:

$$\text{ECSA=}\frac{\text{C}_{\text{dl}}}{\text{C}_{\text{s}}}\text{ (1)}$$

The natural seawater was collected from coastal area in Yellow Sea, Rizhao, Shandong, China. The collected natural seawater was first filtered multiple times through alkaline filter paper to remove the particulate pollutants (e.g., sediment and algae), which will eliminate the influence of impurities to the electrocatalytic process. Since there are interfering ions such as Mg^2+^ and Ca^2+^ in the natural seawater that can affect the catalytic capability of the catalysts, the seawater has been treated with 1.0 M KOH, then centrifuged and filtered to eliminate the influence of sediment and interfering ions.

**1.5 Anion-exchange-membrane water electrolysis (AEM-WE) electrolyzer**

The AEMWE electrolyzer with a serpentine flow channel and an effective area of 0.6 cm^2^ was used to evaluate the application prospect of the FeO_x_-ACs/Co_x_P in the practical water electrolysis. The FeO_x_-ACs/Co_x_P was employed as cathodic catalyst, while the prepared commercial Pt/C (Com Pt/C) was used as anodic catalyst. The anion exchange membrane was immersed into 1.0 M KOH solution for at least 24 h prior to being used to exchange Cl^-^ into OH^-^. Hydrophilic carbon paper (CP) was used as a cathode porous current collector. The Com Pt/C catalyst ink were sprayed onto the CP using an air spray gun, and the total catalyst loading amount is about 2.0 mg·cm^-2^. A titanium felt was used as the anode porous collector. The FeO_x_-ACs/Co_x_P catalyst ink was sprayed onto the titanium felt using an air spray gun, and the total catalyst loading was approximately 2.0 mg·cm^-2^. The anion exchange membrane was sandwiched between the FeO_x_-ACs/Co_x_P-coated titanium felt and Com Pt/C-coated CP. All the AEMWE electrolyzers were operated at 60°C with a peristaltic pump pumping 1.0 M KOH at a flow rate of 5 mL·min^-1^. Polarization curves were obtained from 1.2-2.4 V at a scan rate of 5 mV s^-1^. The stability test of the Pt/C || FeO_x_-ACs/Co_x_P was conducted by measuring the voltage at a current density of 500 mA·cm^-2^ operated at 60°C in 1.0 M KOH solution.

**Section S2 Computational methods**

Density functional theory (DFT) calculations used the Vienna Ab initio Simulation Package (VASP) to calculate the binding energy for specific surfaces of catalysts. The Perdew, Burke and Ernzerhof (PBE) parameterized generalized exchange-correlation interactions are implemented by the VASP package. A cutoff energy of 400 eV was used in all calculations. The Brillouin zones were sampled by k-point meshes, with grid spacing of 2π × 0.01 Å^-1^ or less for all geometric optimization and DOS of bulk, and 2 × 2 × 1 k-point grid was used to calculate all slab models. For all theoretical models, the convergence threshold was set as 10^-5^ eV in energy and 0.01 eV Å^-1^ in force. The correction of van der Waals interaction was included using the DFT-D2 method.

**Section S3 Supplementary Figures and Tables**

**
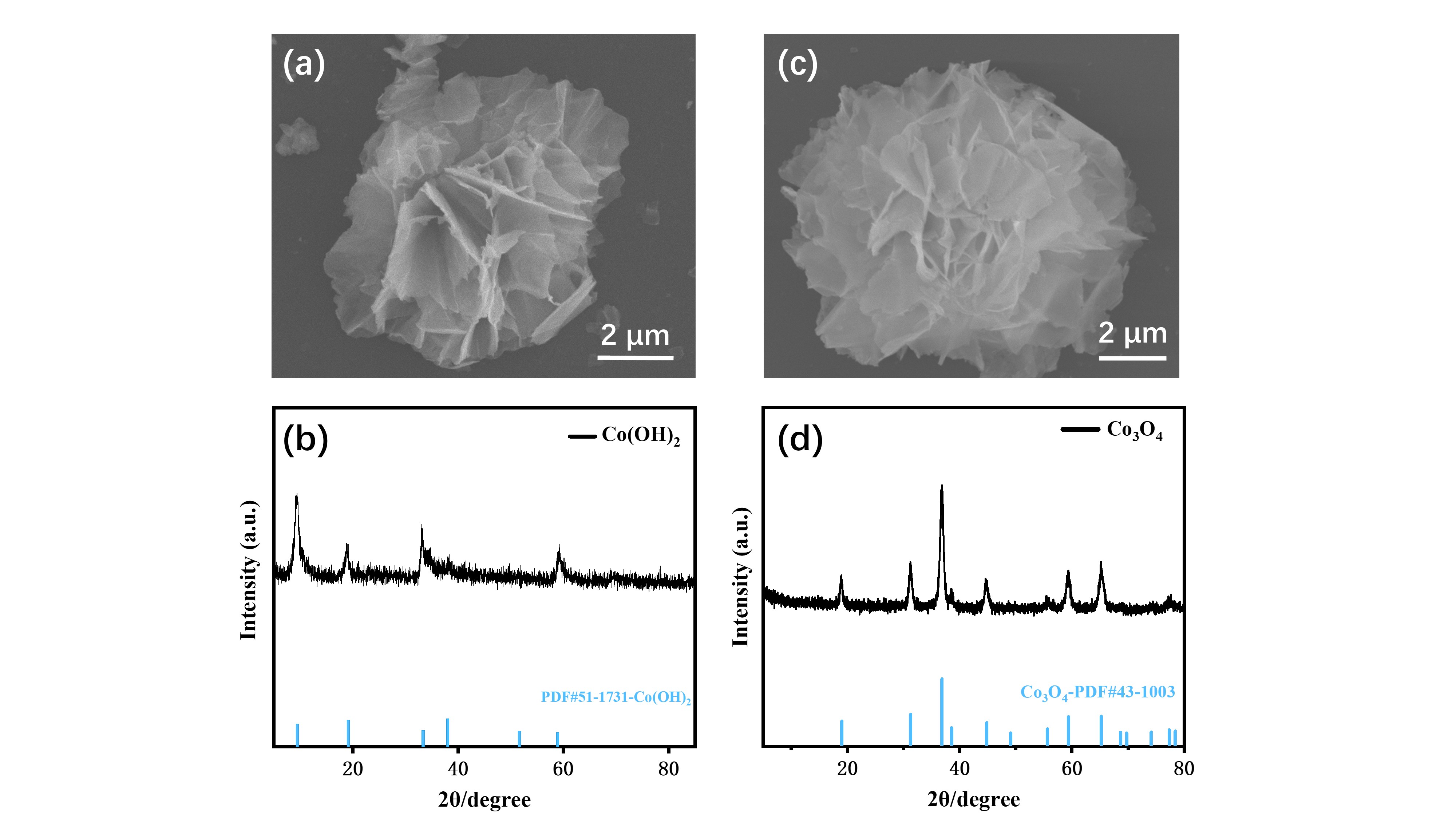
**

**Figure S1.** (a) SEM image and (b) XRD spectrum of Co(OH)_2_. (c) SEM image and (b) XRD spectrum of Co_3_O_4_.





**Figure S2.** XRD spectra of Fe-CoO.





**Figure S3.** (a) SEM image of Co_3_O_4_. (b) HR-TEM images of Fe-CoO. (c) SAED patterns of Fe-CoO.





**Figure S4.** (a-d) EDS element mapping of Fe-CoO.





**Figure S5.** FT-IR spectra of FeO_x_-ACs/Co_x_P.





**Figure S6.** (a) TEM image of FeO_x_-ACs/Co_x_P. (b-c) Partial magnification of the TEM image of FeO_x_-ACs/Co_x_P.





**Figure S7.** HR-TEM images of FeO_x_-ACs/Co_x_P.





**Figure S8.** SEM image of Co_x_P.





**Figure S9.** (a-b) HR-TEM images of Co_x_P. (c) SAED patterns of Co_x_P.





**Figure S10.** (a-d) EDS element mapping of Co_x_P.





**Figure S11.** N_2_ adsorption/desorption isotherm of (a) FeO_x_-ACs/Co_x_P, (b) Co_x_P and (c) Fe-CoO. The inset shows the pore size distribution of FeO_x_-ACs/Co_x_P, Co_x_P and Fe-CoO.





**Figure S12.** Survey XPS spectra of FeO_x_-ACs/Co_x_P, Co_x_P and Fe-CoO.





**Figure S13.** High-resolution XPS spectra of the O 1s of FeO_x_-ACs/Co_x_P.





**Figure S14.** (a) Full survey spectrum of Fe-CoO. High-resolution XPS spectra of (b) Fe 2p, (c) Co 2p and (d) O 1s of Fe-CoO.


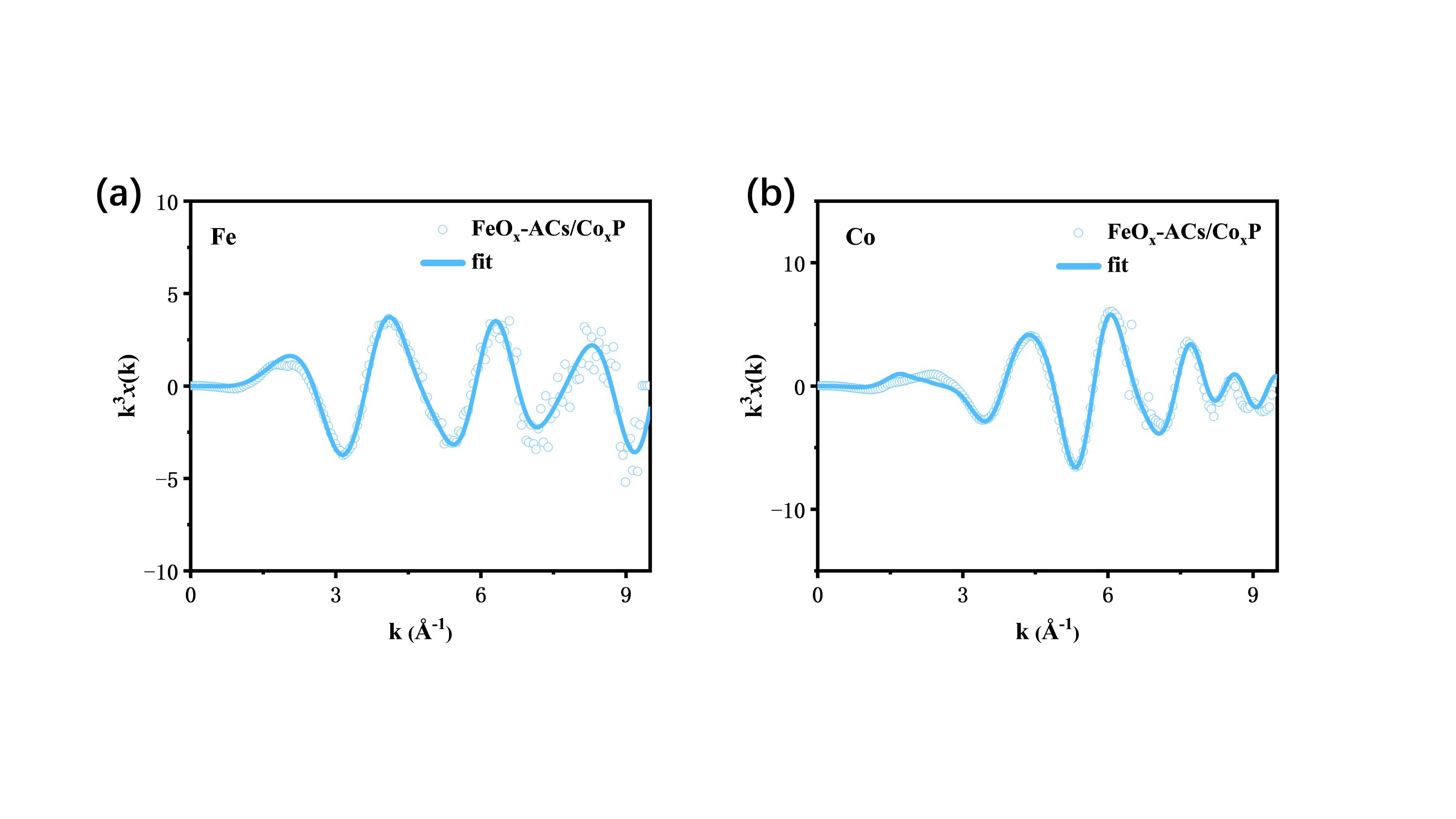


**Figure S15.** (a) The fitting of FT K-space Fe K-edge and (b) Co K-edge EXAFS of FeO_x_-ACs/Co_x_P.


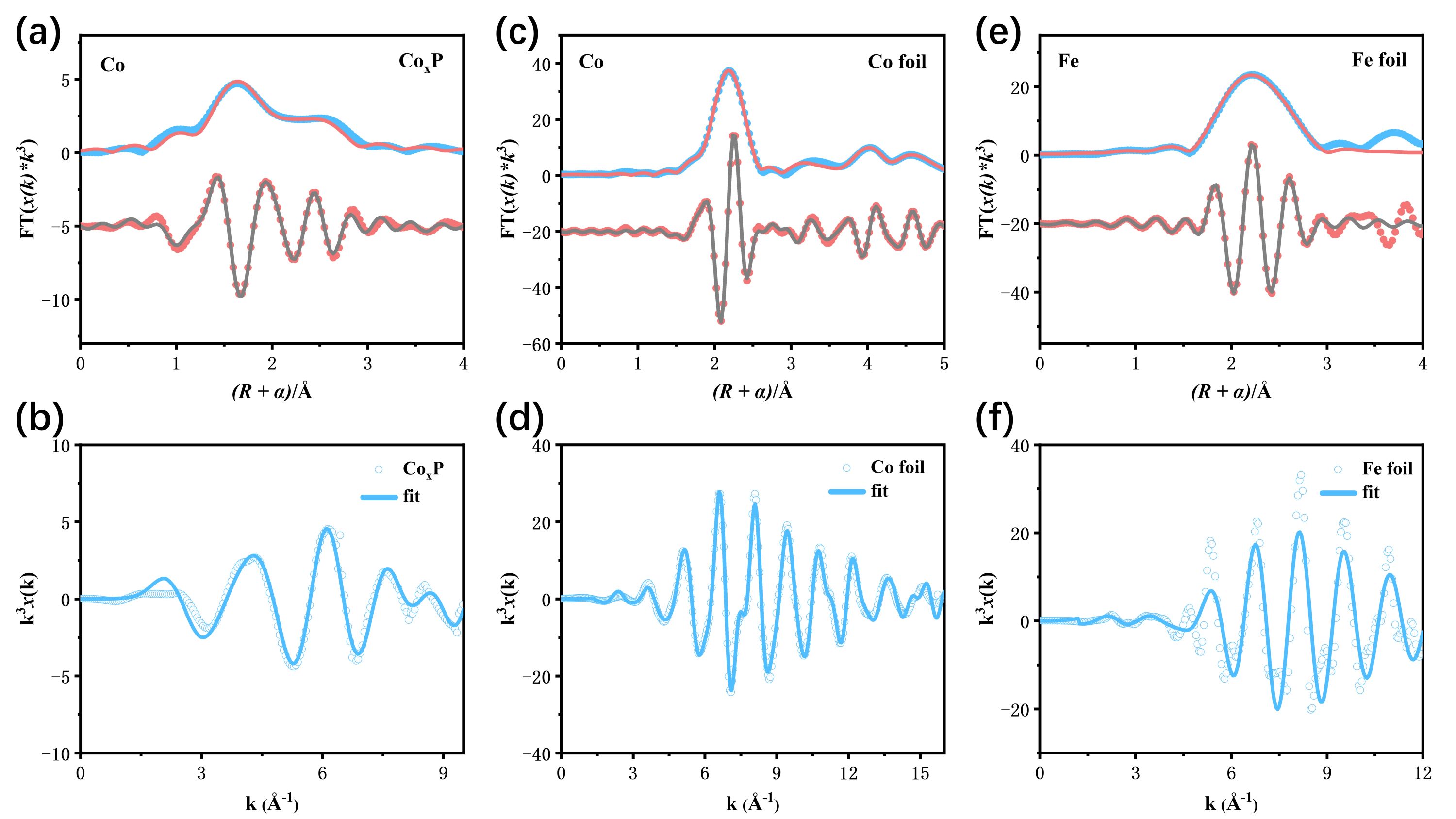


**Figure S16.** (a) The fitting of FT R-space and (b) K-space Co K-edge EXAFS of Co_x_P. (c) The fitting of FT R-space and (d) K-space Co K-edge EXAFS of Co foil. (e) The fitting of FT R-space and (f) K-space Fe K-edge EXAFS of Fe foil.


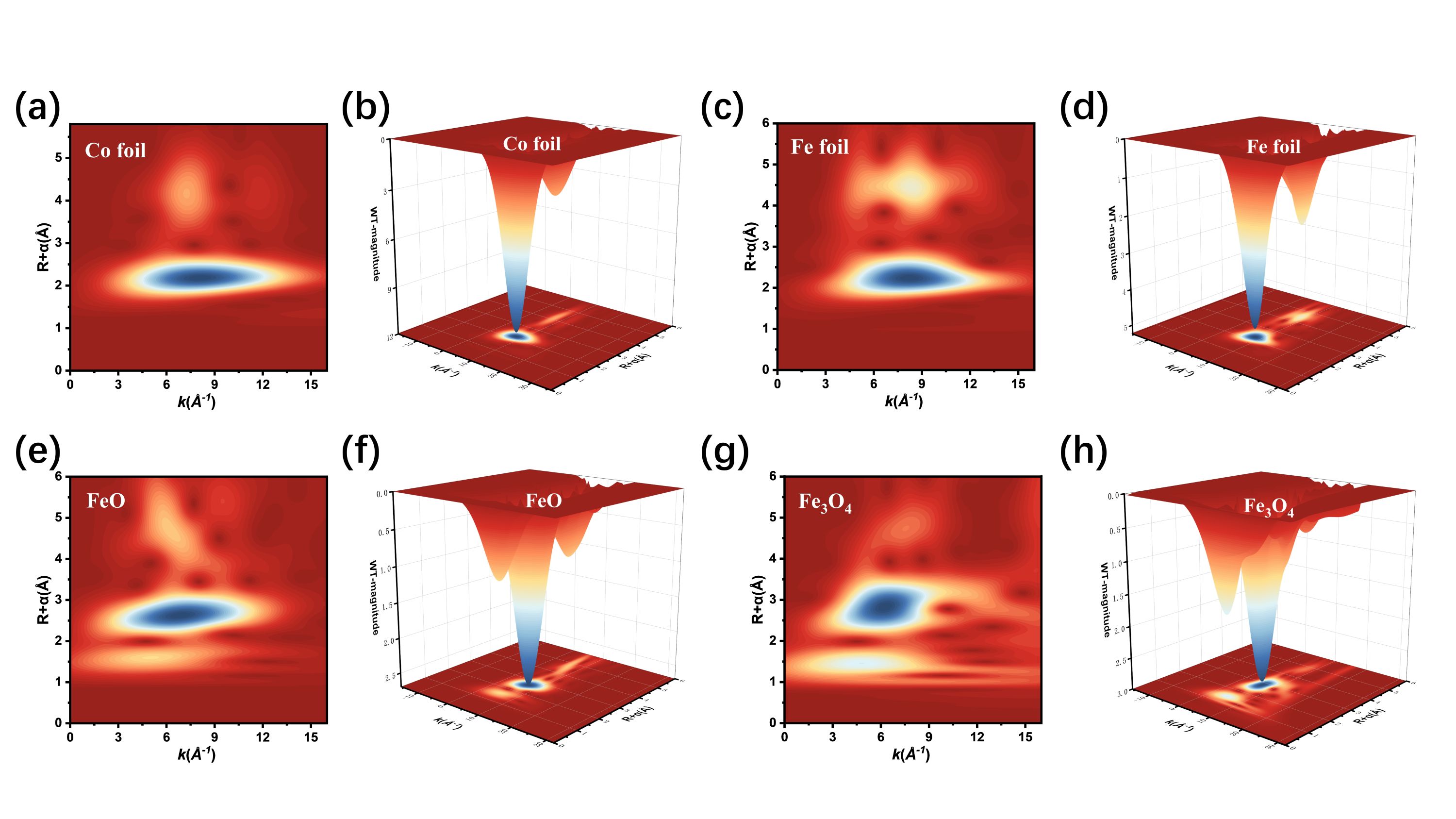


**Figure S17.** (a-b) WT-EXAFS of Co foil at the Co K-edge. (c-d) WT-EXAFS of Fe foil at the Fe K-edge. (e-f) WT-EXAFS of FeO at the Fe K-edge. (g-h) WT-EXAFS of Fe_3_O_4_ at the Fe K-edge.





**Figure S18.** (a) Polarization curves for OER activity of Fe added in different proportions. (b) Polarization curves for OER activity of phosphating in different proportions.





**Figure S19.** Polarization curves for OER activity of FeO_x_-ACs/Co_x_P in high current.





**Figure S20.** CV curves of (a) Co_x_P (b) Fe-CoO (c) Commercial RuO_2_ and (d) Co_3_O_4_ recorded at different scan rates.


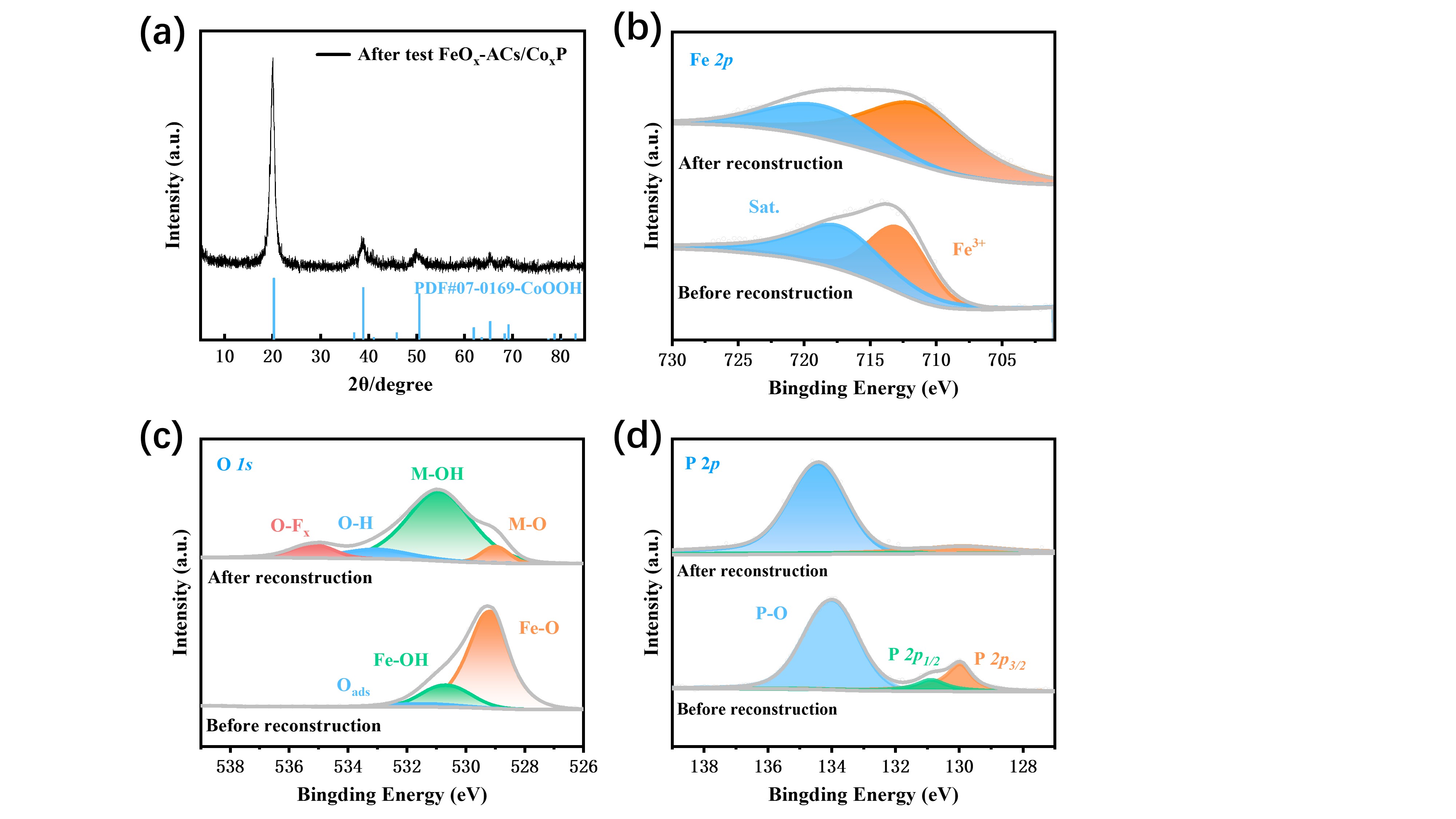


**Figure S21.** (a) XRD spectrum of FeO_x_-ACs/Co_x_P after OER test. The XPS results of FeO_x_-ACs/Co_x_P before and after OER long term stability test. (b) Fe 2p spectrums, (c) O 1s spectrums and (d) P 2p spectrums.





**Figure S22.** The XPS results of Co_x_P before and after OER long term stability test. (a) Co 2p spectrums and (b) P 2p spectrums.





**Figure S23.** *Operando* ATR-SEIRAS FTIR spectroscopic investigation of Co_x_P.





**Figure S24.** LSV curves of (a) Co_x_P and (b) Fe-CoO in 1.0 M KOH and 1.0 M TMAOH, respectively.





**Figure S25.** (a-d) CoOOH structure model, and (e-h) Fe–CoOOH structure model. Color code: blue denotes Co, orange denotes Fe, red denotes O, and white denotes H.


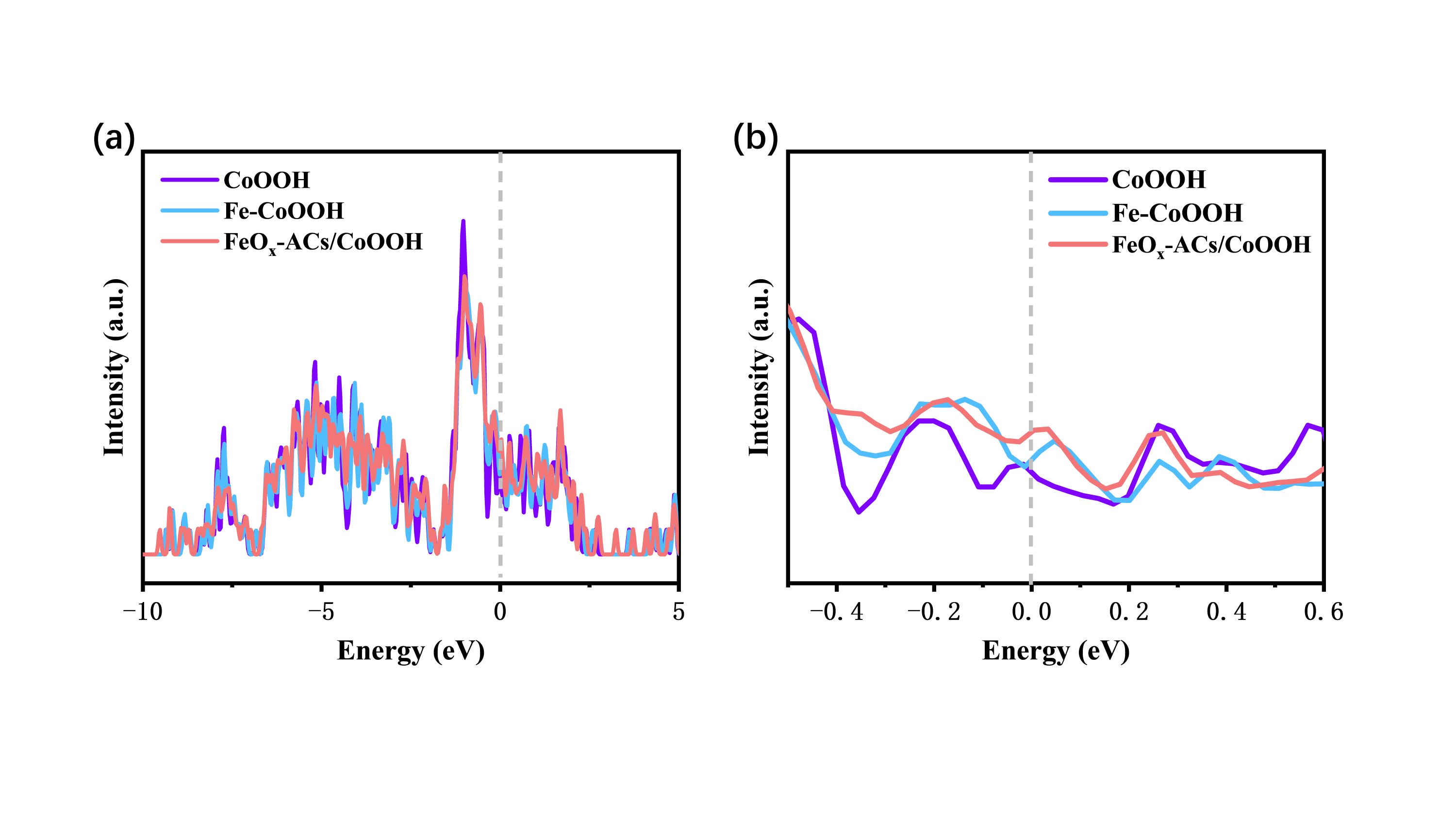


**Figure S26.** (a) The density of states (DOS) of CoOOH, Fe-CoOOH and FeO_x_-ACs/CoOOH. (b) The enlarged DOS diagram of CoOOH, Fe-CoOOH and FeO_x_-ACs/CoOOH around the Fermi level.





**Figure S27.** Comparison of the overpotential among FeO_x_-ACs/Co_x_P and other reported catalysts at 100 mA cm^-2^ in 1.0 M KOH + natural seawater for OER.

**Table S1 ICP-OES**

| **Sample** | **Mass (g)** | **^*1^V_0_** | **Element** | **^*2^C_o_(mg/L)** | **^*3^C_1_(mg/L)** | **^*4^C_x_(mg/kg)** | **^*5^W (%)** |
| --- | --- | --- | --- | --- | --- | --- | --- |
| FeO_x_-ACs/Co_x_P | 0.0237 | 25 | **Fe** | 2.240 | 22.401 | 23629.465 | **2.36** |
|  | 0.0237 | 25 | Co | 3.761 | 376.125 | 396756.461 | 39.68 |
|  | 0.0237 | 25 | P | 21.807 | 218.069 | 230030.361 | 23.00 |

*1: V_o_ refers to the constant volume (ml).

*2: C_o_ refers to the concentration of the element in the test solution.

*3: C_1_ refers to the element concentration in the digested solution/original sample solution.

*4: C_x_ refers to the element content in the sample.

*5: W refers to the element content in the sample.

**Table S2. Curvefit Parameters a for Co K-edge EXAFS for FeO_x_-ACs/Co_x_P.**

| Path | *d* / Å | *N* | *R* / Å | σ^2^ / Å^2^ | R factor |
| --- | --- | --- | --- | --- | --- |
| Co-P | 2.33 | 3.2 | 2.24(±0.02) | 0.01 | 0.02 |
| Co-Co  Co-O | 2.58  3.68 | 1.1  0.3 | 2.60(±0.01)  3.65(±0.01) | 0.02  0.02 |  |

^a^ *S_0_*^2^ is the amplitude reduction factor (*S_0_*^2^ =1). Δ*E_0_* was refined as a global fit parameter. Data ranges: 3.0 ≤ *k* ≤ 9.5 Å^-1^, 1.0 ≤ *R* ≤ 5.0 Å. R: bond distance; σ^2^: Debye-Waller factors; R factor: goodness of fit. R factor for this fit is 2.0 %.

**Table S3. Curvefit Parameters a for Co K-edge EXAFS for Co_x_P.**

| Path | *d* / Å | *N* | *R* / Å | σ^2^ / Å^2^ | R factor |
| --- | --- | --- | --- | --- | --- |
| Co-P | 2.33 | 5.8 | 2.61(±0.02) | 0.01 | 0.02 |
| Co-Co | 2.58 | 0.9 | 2.63(±0.02) | 0.01 |  |

^a^ *S_0_*^2^ is the amplitude reduction factor (*S_0_*^2^ =1). Δ*E_0_* was refined as a global fit parameter. Data ranges: 3.0 ≤ *k* ≤ 10.1 Å^-1^, 1.0 ≤ *R* ≤ 4.8 Å. R: bond distance; σ^2^: Debye-Waller factors; R factor: goodness of fit. R factor for this fit is 1.9 %.

**Table S4.** Curvefit Parameters ^a^ for Co K-edge EXAFS for Co foil.

| Path | *d* / Å | *N* | *R* / Å | σ^2^ / Å^2^ | R factor |
| --- | --- | --- | --- | --- | --- |
| Co-Co | 2.484 | 12 ^b^ | 2.49(±0.003) | 0.006 | 0.01 |
| Co-Co1 | 3.513 | 6 ^b^ | 3.50(±0.01) | 0.01 |  |
| Co-Co2 | 4.303 | 24 ^b^ | 4.34(±0.01) | 0.007 |  |

^a^ *S_0_*^2^ is the amplitude reduction factor (*S_0_*^2^ =1). Δ*E_0_* was refined as a global fit parameter. Data ranges: 3.0 ≤ *k* ≤ 16.0 Å^-1^, 1.0 ≤ *R* ≤ 6.0 Å. R: bond distance; σ^2^: Debye-Waller factors; R factor: goodness of fit. R factor for this fit is 1.0 %. ^b^ These coordination numbers were constrained as *N*(Co-Co) = 12, *N*(Co-Co1) = 6 and *N*(Co-Co2) = 24 based on the crystal structure of Co foil.

**Table S5. Curvefit Parameters a for Fe K-edge EXAFS for FeO_x_-ACs/Co_x_P.**

| Path | *d* / Å | *N* | *R* / Å | σ^2^ / Å^2^ | R factor |
| --- | --- | --- | --- | --- | --- |
| Fe-O1  Fe-O2 | 1.966  2.122 | 4.5  0.1 | 1.99(±0.02)  2.00(±0.02) | 0.01  0.01 | 0.02 |
| Fe-Fe | 3.00 | 3.1 | 3.03(±0.02) | 0.02 |  |

^a^ *S_0_*^2^ is the amplitude reduction factor (*S_0_*^2^ =1). Δ*E_0_* was refined as a global fit parameter. Data ranges: 3.0 ≤ *k* ≤ 9.5 Å^-1^, 1.0 ≤ *R* ≤ 4.7 Å. R: bond distance; σ^2^: Debye-Waller factors; R factor: goodness of fit. R factor for this fit is 2.2 %.

**Table S6.** Curvefit Parameters ^a^ for Fe K-edge EXAFS for Fe foil.

| Path | *d* / Å | *N* | *R* / Å | σ^2^ / Å^2^ | R factor |
| --- | --- | --- | --- | --- | --- |
| Fe-Fe | 2.458 | 8 ^b^ | 2.48(±0.003) | 0.002 | 0.01 |
| Fe-Fe1 | 2.814 | 6 ^b^ | 2.86(±0.01) | 0.001 |  |

^a^ *S_0_*^2^ is the amplitude reduction factor (*S_0_*^2^ =1). Δ*E_0_* was refined as a global fit parameter. Data ranges: 3.0 ≤ *k* ≤ 10.2 Å^-1^, 1.0 ≤ *R* ≤ 3.2 Å. R: bond distance; σ^2^: Debye-Waller factors; R factor: goodness of fit. R factor for this fit is 1.7 %. ^b^ These coordination numbers were constrained as *N*(Fe-Fe) = 8 and *N*(Fe-Fe1) = 6 based on the crystal structure of Fe foil.

**Table S7**

| **Materials** | **Tafel slope (mV dec^-1^)** | **Overpotential (mV@100 mA cm^-2^)** | ***Ref.*** |
| --- | --- | --- | --- |
| **This work** | **30.01** | **278** |  |
| F-CDs/CoP/NF | 96 | 328 | *Ref.* ^[1]^ |
| NiCo-O_xy_-SH | 63 | 350 | *Ref.* ^[2]^ |
| Zn-Fe/Mn@Mn-FeP | 51.9 | 355 | *Ref.* ^[3]^ |
| r-Ru/FeCoP | 45.24 | 320 | *Ref.* ^[4]^ |
| CoP/FeCoP_x_ | 54.6 | 284 | *Ref.* ^[5]^ |
| Al-FeCoP | 28.9 | 422 | *Ref.* ^[6]^ |
| NiCoP-NPCNT-NF | 112.9 | 380 | *Ref.* ^[7]^ |
| P-CoMo_2_S_4_/Co_4_S_3_-Co_2_P | 54 | 311 | *Ref.* ^[8]^ |
| Fe-P-CMO | 43.4 | 278 | *Ref.* ^[9]^ |
| FeSe/Co_2_P/NF | 65.6 | 420 | *Ref.* ^[10]^ |
| CuO@FeCoP/CF | 89.37 | 313 | *Ref.* ^[11]^ |
| F-FeCoP_v_@IF | 67 | 280 | *Ref.* ^[12]^ |
| N-FeCoP | 48 | 410 | *Ref.* ^[13]^ |
| Ru@FeCoP | 45.7 | 314 | *Ref.* ^[14]^ |
| NMCP@NF | 41 | 320 | *Ref.* ^[15]^ |
| Fe-Ni_x_PO_4_ | 44.89 | 292 | *Ref.* ^[16]^ |
| Fe_x_Ni_y_-LDH/NF | 49.5 | 334 | *Ref.* ^[17]^ |
| FeOOH/FeNiCo-LDH | 32.4 | 299 | *Ref.* ^[18]^ |
| Gss-Co_2_P/CoP@C-800 | 66.7 | 365 | *Ref.* ^[19]^ |

**Table S8**

| **Materials** | **Overpotential (mV@100 mA cm^-2^)** | ***Ref.*** |
| --- | --- | --- |
| **This work** | **298** |  |
| S-(Ni,Fe)OOH | 300 | *Ref.* ^[20]^ |
| Ni_2_P-Fe_2_P | 305 | *Ref.* ^[21]^ |
| NiMoN@NiFeN | 307 | *Ref.* ^[22]^ |
| Cr-Co_x_P | 334 | *Ref.* ^[23]^ |
| Co_1.98_-NiFe LDH | 335 | *Ref.* ^[24]^ |
| Ni_3_S_2_/Co_3_S_4_ | 360 | *Ref.* ^[25]^ |

**References**

[1] H. Song, J. Yu, Z. Tang, B. Yang, S. Lu, *Adv. Energy Mater.* **2022**, *12*, 2102573.

[2] X. Zheng, Y. Cao, Z. Wu, W. Ding, T. Xue, J. Wang, Z. Chen, X. Han, Y. Deng, W. Hu, *Adv. Energy Mater.* **2022**, *12*, 2103275.

[3] L. Huang, R. Yao, X. Wang, S. Sun, X. Zhu, X. Liu, M. G. Kim, J. Lian, F. Liu, Y. Li, H. Zong, S. Han, X. Ding, *Energy Environ. Sci.* **2022**, *15*, 2425.

[4] Y. Wang, X. Chen, Y. Du, S. Li, M. Wang, Y. Yang, L. Wang, *Nanoscale* **2024**, *16*, 3474.

[5] J. Zhang, Y. Zhang, J. Zhou, H. Guo, L. Qi, *Small Methods* **n.d.**, *n/a*, 2401139.

[6] J. He, Y. Wang, L. Mu, J. Li, Y. Dong, Y. Zhang, Y. Deng, P. Feng, M. Qu, *J. Water Process Eng.* **2024**, *68*, 106302.

[7] H. Zhang, H. Guo, Y. Zhang, J. Zhao, Y. Li, X. Li, J. Ren, R. Song, *ACS Sustain. Chem. Eng.* **2022**, *10*, 6402.

[8] K. Dong, D. T. Tran, X. Li, S. Prabhakaran, D. H. Kim, N. H. Kim, J. H. Lee, *Appl. Catal. B Environ. Energy* **2024**, *344*, 123649.

[9] B. Wang, X. Chen, Y. He, Q. Liu, X. Zhang, Z. Luo, J. V. Kennedy, J. Li, D. Qian, J. Liu, G. I. N. Waterhouse, *Appl. Catal. B Environ. Energy* **2024**, *346*, 123741.

[10] S. Liu, Y. Xing, Z. Zhou, Y. Yang, Y. Li, X. Xiao, C. Wang, *J. Mater. Chem. A* **2023**, *11*, 8330.

[11] S. Jiang, Q. Li, Q. Zhao, L. Cheng, T. Jiang, *J. Alloys Compd.* **2025**, *1010*, 177291.

[12] J. Zhu, J. Chi, T. Cui, L. Guo, S. Wu, B. Li, J. Lai, L. Wang, *Appl. Catal. B Environ.* **2023**, *328*, 122487.

[13] Z. Liu, T. Zhang, Y. Lin, H. Jia, Y. Wang, Y. Wang, G. Zhang, *Small* **2023**, *19*, 2302475.

[14] Y. Wang, Y. Du, Z. Fu, J. Ren, Y. Fu, L. Wang, *J. Mater. Chem. A* **2022**, *10*, 16071.

[15] M. R. Kandel, U. N. Pan, D. R. Paudel, P. P. Dhakal, N. H. Kim, J. H. Lee, *Compos. Part B Eng.* **2022**, *239*, 109992.

[16] D. Xu, C. Zhang, Y. Liu, Q. Ye, J. Lu, Y. Zhao, J. Ma, Y. Cheng, *Small* **n.d.**, *n/a*, 2410739.

[17] R. M. Bhattarai, L. Nguyen, N. Le, K. Chhetri, D. Acharya, S. Teke, S. Saud, D. B. Nguyen, S. J. Kim, Y. S. Mok, *Small* **n.d.**, *n/a*, 2410027.

[18] C. Zhang, F. Li, D. Wu, Q. Guo, Z. Liu, Z. Wang, Z. Kang, L. Fan, D. Sun, *Inorg. Chem. Front.* **2025**, DOI 10.1039/D4QI03103B.

[19] Y. Luo, Y. Yang, Y. Tian, Q. Wu, W.-F. Lin, M. Wen, *J. Mater. Chem. A* **2025**, DOI 10.1039/D4TA08586H.

[20] L. Yu, L. Wu, B. McElhenny, S. Song, D. Luo, F. Zhang, Y. Yu, S. Chen, Z. Ren, *Energy Environ. Sci.* **2020**, *13*, 3439.

[21] L. Wu, L. Yu, F. Zhang, B. McElhenny, D. Luo, A. Karim, S. Chen, Z. Ren, *Adv. Funct. Mater.* **2021**, *31*, DOI 10.1002/adfm.202006484.

[22] L. Yu, Q. Zhu, S. Song, B. McElhenny, D. Wang, C. Wu, Z. Qin, J. Bao, Y. Yu, S. Chen, Z. Ren, *Nat. Commun.* **2019**, *10*, DOI 10.1038/s41467-019-13092-7.

[23] Y. Song, M. Sun, S. Zhang, X. Zhang, P. Yi, J. Liu, B. Huang, M. Huang, L. Zhang, *Adv. Funct. Mater.* **2023**, *33*, DOI 10.1002/adfm.202214081.

[24] Y. Yang, S. Wei, Y. Li, D. Guo, H. Liu, L. Liu, *Appl. Catal. B Environ.* **2022**, *314*, 121491.

[25] C. Wang, M. Zhu, Z. Cao, P. Zhu, Y. Cao, X. Xu, C. Xu, Z. Yin, *Appl. Catal. B Environ.* **2021**, *291*, 120071.
